# Oroxylin A promotes PTEN-mediated negative regulation of MDM2 transcription via SIRT3-mediated deacetylation to stabilize p53 and inhibit glycolysis in wt-p53 cancer cells

**DOI:** 10.1186/s13045-015-0137-1

**Published:** 2015-04-23

**Authors:** Kai Zhao, Yuxin Zhou, Chen Qiao, Ting Ni, Zhiyu Li, Xiaotang Wang, Qinglong Guo, Na Lu, Libin Wei

**Affiliations:** State Key Laboratory of Natural Medicines, Jiangsu Key Laboratory of Carcinogenesis and Intervention, China Pharmaceutical University, 24 Tongjiaxiang, Nanjing, 210009 The People’s Republic of China; Department of Medicinal Chemistry, China Pharmaceutical University, 24 Tongjiaxiang, Nanjing, 210009 The People’s Republic of China; Department of Chemistry and Biochemistry, Florida International University, Miami, FL 33199 USA

**Keywords:** Oroxylin A, Glycolysis, MDM2, PTEN, SIRT3

## Abstract

**Introduction:**

p53 plays important roles in regulating the metabolic reprogramming of cancer, such as aerobic glycolysis. Oroxylin A is a natural active flavonoid with strong anticancer effects both *in vitro* and *in vivo*.

**Methods:**

wt-p53 (MCF-7 and HCT116 cells) cancer cells and p53-null H1299 cancer cells were used. The glucose uptake and lactate production were analyzed using Lactic Acid production Detection kit and the Amplex Red Glucose Assay Kit. Then, the protein levels and RNA levels of p53, mouse double minute 2 (MDM2), and p53-targeted glycolytic enzymes were quantified using Western blotting and quantitative polymerase chain reaction (PCR), respectively. Immunoprecipitation were performed to assess the binding between p53, MDM2, and sirtuin-3 (SIRT3), and the deacetylation of phosphatase and tensin homolog (PTEN). Reporter assays were performed to assess the transcriptional activity of PTEN. *In vivo*, effects of oroxylin A was investigated in nude mice xenograft tumor-inoculated MCF-7 or HCT116 cells.

**Results:**

Here, we analyzed the underlying mechanisms that oroxylin A regulated p53 level and glycolytic metabolism in wt-p53 cancer cells, and found that oroxylin A inhibited glycolysis through upregulating p53 level. Oroxylin A did not directly affect the transcription of wt-p53, but suppressed the MDM2-mediated degradation of p53 via downregulating MDM2 transcription in wt-p53 cancer cells. In further studies, we found that oroxylin A induced a reduction in MDM2 transcription by promoting the lipid phosphatase activity of phosphatase and tensin homolog, which was upregulated via sirtuin3-mediated deacetylation. *In vivo*, oroxylin A inhibited the tumor growth of nude mice-inoculated MCF-7 or HCT116 cells. The expression of MDM2 protein in tumor tissue was downregulated by oroxylin A as well.

**Conclusions:**

These results provide a p53-independent mechanism of MDM2 transcription and reveal the potential of oroxylin A on glycolytic regulation in both wt-p53 and mut-p53 cancer cells. The studies have important implications for the investigation on anticancer effects of oroxylin A, and provide the academic basis for the clinical trial of oroxylin A in cancer patients.

**Electronic supplementary material:**

The online version of this article (doi:10.1186/s13045-015-0137-1) contains supplementary material, which is available to authorized users.

## Introduction

p53 gene is one of the most highly studied tumor suppressors, and is often considered as the ‘cellular gatekeeper’ [1]. By responding to the constant bombardment of various stresses against cell survival, p53 functions diligently and faithfully, promoting cell cycle arrest, apoptosis, cellular senescence, or differentiation through different mechanisms [2]. p53 acts as a critical ‘node’ in the cellular circuitry, yet it is mutated in over 50% of all human tumors, which makes the development of novel anticancer drugs targeting p53 difficult [3]. As the first tumor suppressor gene is shown to be involved in the regulation of tumor metabolism, p53 plays important roles in metabolic regulation, in addition to its established roles in cell survival and apoptosis.

The metabolic changes that occur in cancer cells have been known for decades; however, the complexity and importance of those changes have only been understood in recent years. The metabolic switch from oxidative phosphorylation to aerobic glycolysis facilitates the growth of cancer cells. By regulating the levels of a series of gene products that affect metabolic fates and metabolic products, p53 helps to slow down glycolysis and promote oxidative phosphorylation [4]. For example, p53 upregulates the expression of synthesis of cytochrome c oxidase 2 (SCO2) and TP53-induced glycolysis and apoptosis regulator (TIGAR), whereas it downregulates the expression of phosphoglycerate mutase (PGM) and glucose transporters 1 and 4 (GLUT1, GLUT4) [5-8]. Therefore, many drugs targeting energy metabolism are in development.

p53 modulates many key glycolytic enzymes as a specific transcription factor. The activity of p53 is significant and highly regulated by post-translational modifications, protein-protein interactions, and protein stabilization. In unstressed cells, p53 levels are kept low through its continuous degradation. Mouse double minute 2 (MDM2), the predominant negative regulator of p53, normally maintains p53 at low levels. MDM2 (also called HDM2 in humans), the expression of which is regulated by p53, can bind to and inactivate p53, transporting it from the nucleus to the cytosol. MDM2 also functions as ubiquitin ligase and covalently attaches ubiquitin to p53, marking the protein for degradation by the proteasome [9]. Several mechanisms are activated to promote the rapid accumulation of p53, including the post-translational modification of p53 and MDM2, subcellular redistribution, inhibition of MDM2 activity, and direct repression of MDM2 transcription [10].

Sirtuin-3 (SIRT3) is a member of the SIRT family of proteins, which are class III NAD + -dependent histone deacetylases that are involved in a variety of functions including the regulation of metabolism, aging, and carcinogenesis [11]. SIRT3, as a mitochondrial tumor suppressor protein, is responsible for several actions that depend on its mitochondrial milieu, including the considerable deacetylation of mitochondrial proteins, along with a decrease of glycolysis and ATP levels [12]. In addition to its reported mitochondrial function, the existence of a small pool of active nuclear SIRT3 has been proposed. This pool consists of the long form of SIRT3 and has been suggested to have histone deacetylase activity [13].

In the previous studies, we showed that oroxylin A (OA), a flavonoid isolated from scutellaria root, inhibited cell growth and induced apoptosis in various cancer cells, such as human breast cancer MCF-7 and MDA-MB-231 cells [14] and human colon cancer HCT116 and HT29 cells [15,16]. Oroxylin A inhibited glycolysis by promoting the SIRT3-mediated deacetylation of cyclophilin D in breast carcinoma. Moreover, oroxylin A stabilized p53 expression at the post-translational level by downregulating MDM2 expression [17]. It was reported that SIRT3 inhibited cancer cell growth by reducing MDM2-mediated p53 degradation. However, the underlying mechanism remains unclear. Therefore, in the present study, we further investigated the mechanism underlying the regulation of oroxylin A on p53 degradation involved in the SIRT3-mediated deacetylation. We found that oroxylin A remarkably inhibited aerobic glycolysis in wt-p53 cancer cells and suppressed MDM2-mediated degradation of p53 through inhibiting SIRT3-modulated transcription of MDM2. Furthermore, SIRT3 played critical roles in the oroxylin A-induced deacetylation of the phosphatase and tensin homolog (PTEN), resulting in the negative transcription of MDM2. Some other reports also demonstrate that PTEN blocks MDM2 nuclear translocation and destabilizes the MDM2 protein [18]. Taken together, our studies expand the knowledge of the post-transcriptional regulation of MDM2 and reveal a novel mechanism to explain the anticancer effect of oroxylin A.

## Results

### Oroxylin A inhibited p53-regulated glycolysis in wt-p53 cancer cells

**Figure 1:**
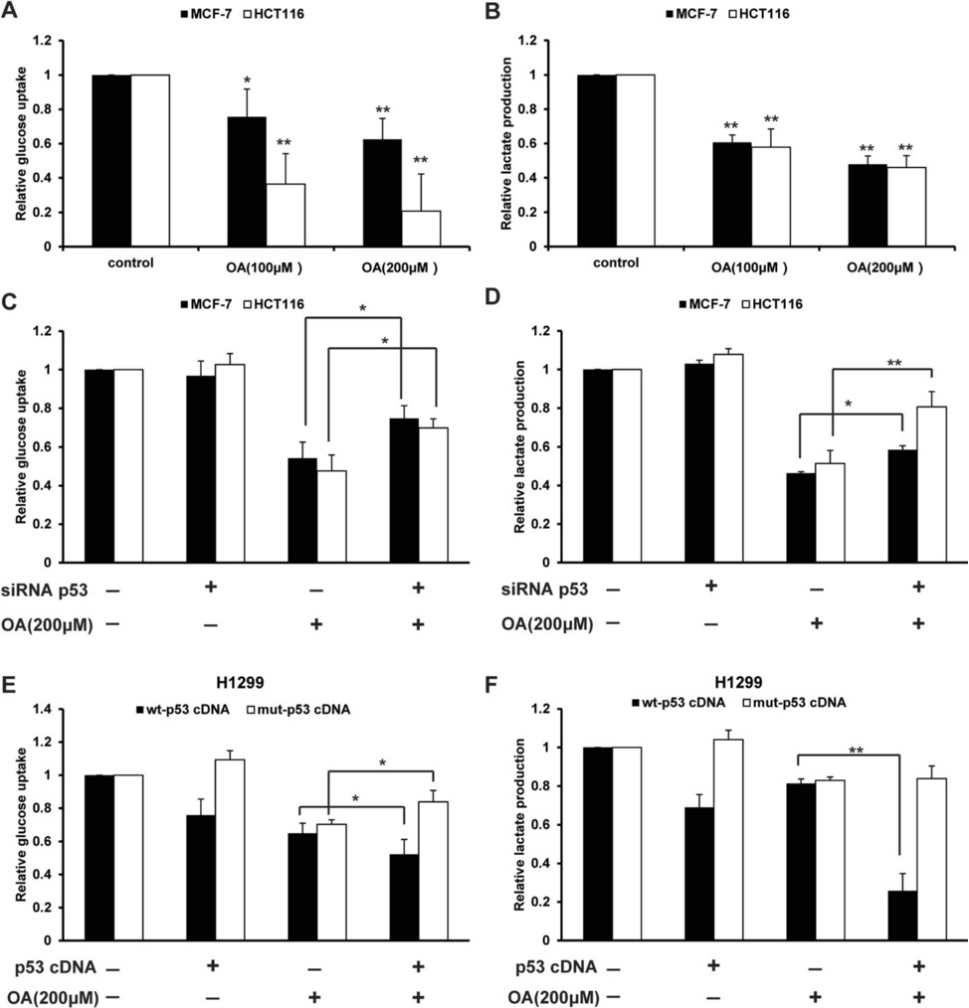
P53 plays an important role in oroxylin A-induced suppression of glycolysis. **(A, B)** Wt-p53 cancer cells (MCF-7 and HCT116) were treated with oroxylin A (100 and 200 μΜ) for 48 h. **(A)** Glucose uptake was measured using the Amplex Red assay. **(B)** Production of lactic acid was assayed by Lactic Acid production Detection kit. **(C, D)** MCF-7 and HCT116 were transfected with siRNA targeting wt-p53 or with a non-targeting control siRNA, then incubated with 200 μM oroxylin A for 48 h. Glucose uptake **(C)** and lactate production **(D)** were detected. **(E, F)** Cells were transfected with a cDNA clone targeting wt-p53, or mut-p53 or with a non-targeting vector. Cells were then incubated with 200 μM oroxylin A for 48 h. Glucose uptake **(E)** and lactate production **(F)** were detected. Bars, SD; *p < 0.05 or **p < 0.01 versus non-treated control.

In previous studies, we found that oroxylin A inhibited the cell growth of wt-p53 cancer cell lines MCF-7 and HCT116 cells. Here, we investigated the influence of oroxylin A on aerobic glycolysis of these cancer cells. Oroxylin A (100 and 200 μΜ) inhibited the glucose uptake and lactate production in MCF-7 and HCT116 cells (Figure 1A, B). Transfection of cells with small interference RNA (siRNA) targeting wt-p53 abolished the inhibitory effects of oroxylin A on glucose uptake and lactate production in wt-p53 cancer cells (Figure 1C, D). To determine the importance of p53 in the glycolytic inhibitory effects of oroxylin A, p53-null H1299 cancer cells were transfected with wt-p53 cDNA or mut-p53 cDNA (R249S). As a result, only the cells expressing wt-p53 cDNA appeared with decreased glycolysis and promoted the inhibitory effects of oroxylin A on glycolysis; the cells transfected with the mut-p53 cDNA were against oroxylin A’s effects and could not reverse the decrease of glucose uptake and lactate generation induced by oroxylin A (Figure 1E, F).

**Figure 2:**
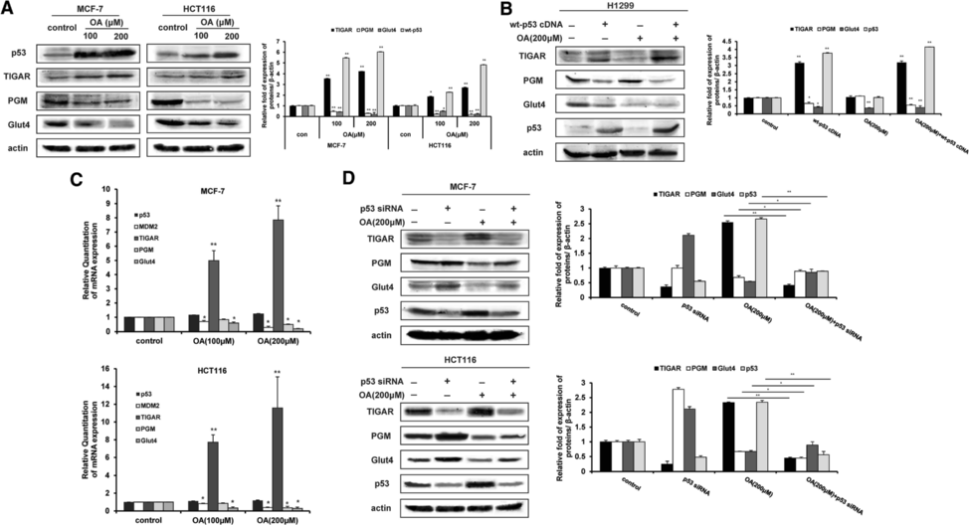
Oroxylin A downregulates the protein and mRNA expression of p53-related glycolytic pathway components. **(A)** MCF-7 and HCT116 cells were treated with oroxylin A (100 and 200 μΜ) for 48 h. Western blot assays were performed for the p53-targeted gene products p53, TIGAR, PGM, and GLUT4. **(B)** H1299 cells were transfected with a cDNA clone targeting wt-p53 or with a non-targeting vector, then incubated with 200 μM oroxylin A for 48 h. Western blot assays were performed for the p53-targeted gene products TIGAR, PGM, and GLUT4. **(C)** The gene expressions of p53, MDM2, and p53-targeted genes were detected by Quantitative RT-PCR. **(D)** MCF-7 and HCT116 were transfected with siRNA targeting wt-p53 or with a non-targeting control siRNA, then incubated with 200 μM oroxylin A for 48 h. Western blot assays were performed for the p53-targeted gene products TIGAR, PGM, and GLUT4. All the Western Blot bands were quantified. Bars, SD; *p < 0.05 or **p < 0.01 versus non-treated control.

Then, we further investigated the effects of oroxylin A on the expression of TIGAR, PGM, and GLUT4, which are target genes of p53 and are responsible for glucose metabolism. As shown in Figure 2A, oroxylin A increased p53 protein level, induced the expression of TIGAR, and inhibited the expressions of PGM and GLUT4 in MCF-7 and HCT116 cells. Transfection of H1299 cells with wt-p53 cDNA upregulated the protein expression of TIGAR and downregulated the protein expressions of PGM and GLUT4 (Figure 2B). Assessment of the mRNA expression levels of p53 and p53-targeted genes showed that oroxylin A increased the mRNA expression of TIGAR and decreased that of PGM and GLUT4, but had little influence on p53 mRNA level (Figure 2C). Moreover, the deletion of p53 in MCF-7 and HCT116 cells reversed the influence of oroxylin A on p53-targeted protein expressions (Figure 2D).

These results suggested that p53 played important roles in the oroxylin A-induced suppression of glycolysis.

### Oroxylin A inhibited glycolysis in wt-p53 cancer cells through suppressing MDM2-mediated p53 degradation

**Figure 3:**
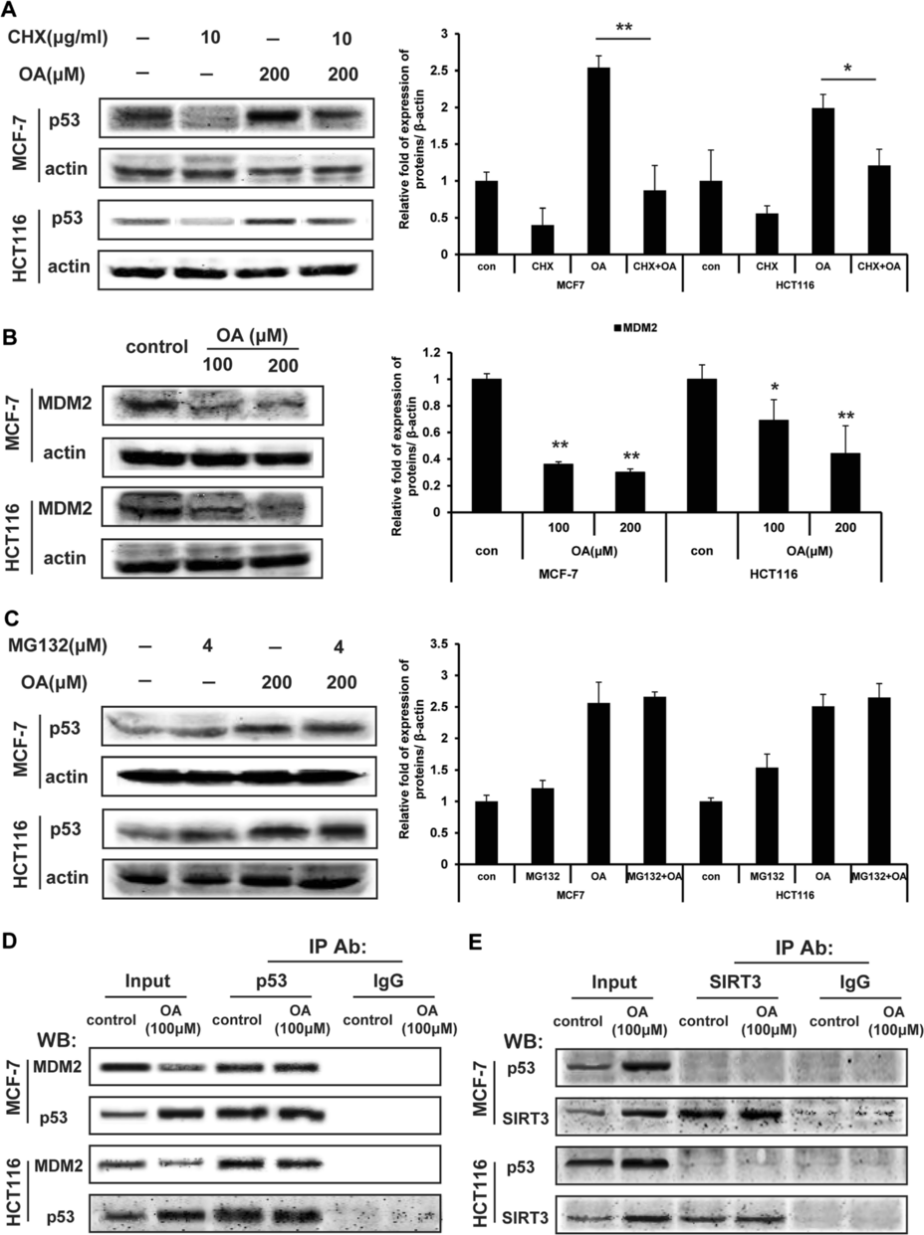
Oroxylin A enhances p53 expression through post-transcriptional regulation. **(A)** Cells were treated with oroxylin A (100 and 200 μΜ) for 48 h. Western blot assays were performed for MDM2. **(B)** Effect of oroxylin A on p53 expression after co-treatment with CHX. Cells were treated with vehicle or oroxylin A for 48 h, and 6 h before harvested, 10 μg/ml CHX was added to the medium. p53 protein expression was detected by Western blotting. **(C)** Effect of oroxylin A on p53 expression after co-treatment with MG132. Cells were treated with oroxylin A for 48 h, and 6 h before harvested, 4 μM MG132 was added to the medium. p53 expression was detected by Western blotting. **(D)** MDM2 was immunoprecipitated using p53 (Ab6) antibodies. Western blot assays were performed for MDM2, p53. **(E)** p53 (Ab6) was immunoprecipitated using anti-SIRT3 antibody. Western blot assays were performed for p53 and SIRT3. All the Western blot bands were quantified.

Based on the above results, oroxylin A increased the p53 protein level in MCF-7 and HCT116 cells, but had little influence on the transcriptional level of p53. Therefore, we used cycloheximide (CHX), an inhibitor of protein synthesis, and found that oroxylin A still upregulated the expression of p53 upon the co-treatment of CHX (Figure 3A). These data further suggested that oroxylin A modulates wt-p53 expression at the post-translational level.

Since MDM2 is critical in promoting wt-p53 degradation via the proteasome pathway, we examined whether MDM2 was involved in the oroxylin A-mediated upregulation of p53. The results showed that oroxylin A decreased MDM2 mRNA and protein levels (Figures 2C and 3B). In the presence of MG132, an inhibitor of proteasome-mediated proteolysis, oroxylin A-increased wt-p53 protein expression was changed minimally (Figure 3C), indicating that oroxylin A increased p53 levels by suppressing MDM2-modulated proteasomal degradation. Moreover, oroxylin A did not affect the binding of wt p53 and MDM2 (Figure 3D).

Besides ubiquitination, phosphorylation and acetylation are post-translational modifications to p53 that have a profound effect on p53 stability and function. In our previous studies, oroxylin A promoted glycolysis in human breast cancer cells by upregulating SIRT3, which is an NAD + -dependent deacetylase. To investigate whether oroxylin A could influence the stability of p53 via SIRT3-mediated acetylation, the binding of SIRT3 with p53 was assessed by co-immunoprecipitation. As shown in Figure 3E, p53 could not bind with SIRT3 directly and oroxylin A had no effect on their binding.

Taken together, these results indicated that oroxylin A inhibited p53 degradation by downregulating MDM2 expression.

### The regulation of p53 by oroxylin A is mediated by SIRT3

**Figure 4:**
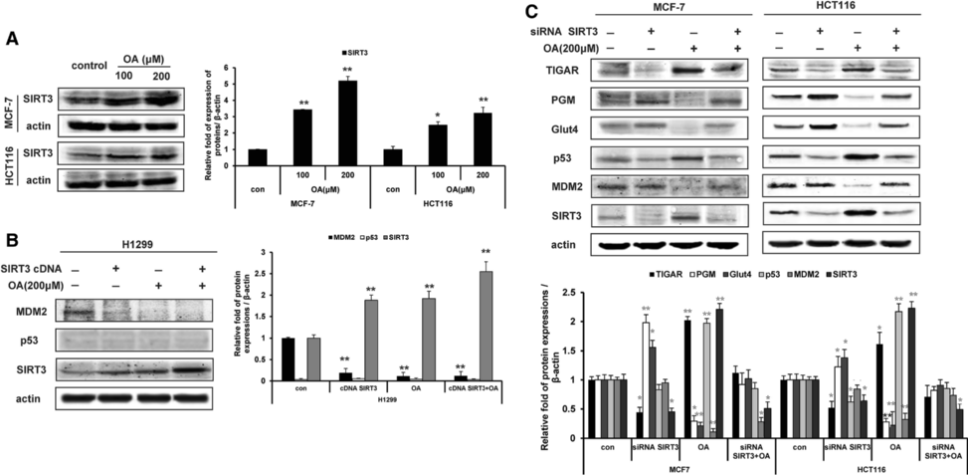
The oroxylin A regulation of p53, MDM2 and p53-related glycolytic pathway components is SIRT3-dependent. **(A)** Cells were treated with oroxylin A (100 and 200 μΜ) for 48 h. Western blot assays were performed for SIRT3. **(B)** H1299 cells were transfected with a cDNA clone targeting SIRT3 or with a non-targeting vector, and then incubated with 200 μM oroxylin A for 48 h. Western blot assays were performed for p53, MDM2, and SIRT3. **(C)** Cells were transfected with siRNA targeting SIRT3 or with a non-targeting control siRNA and incubated with 200 μM oroxylin A for 48 h. Western blot assays were performed for p53, MDM2, and the p53-targeted gene products TIGAR, PGM, and GLUT4. All the Western Blot bands were quantified.

SIRT3 has been shown to inhibit MDM2-mediated p53 degradation [19], and we found that SIRT3 had no direct effects on p53 stability. Therefore, we investigated whether the effect of oroxylin A on p53-related pathways was involved with SIRT3. Oroxylin A increased the protein expression of SIRT3 (Figure 4A). Transfection of cells with SIRT3 cDNA downregulated the expression of MDM2 (Figure 4B) Transfection of MCF-7 and HCT116 cells with siRNA targeting SIRT3 reversed the oroxylin A-induced upregulation of TIGAR and p53 and downregulation of PGM, GLUT4, and MDM2 (Figure 4C). Taken together, these results suggested that the regulation of p53 levels and p53-related pathways by oroxylin A is mediated by SIRT3.

### Oroxylin A downregulates the transcription of MDM2 through PTEN

**Figure 5:**
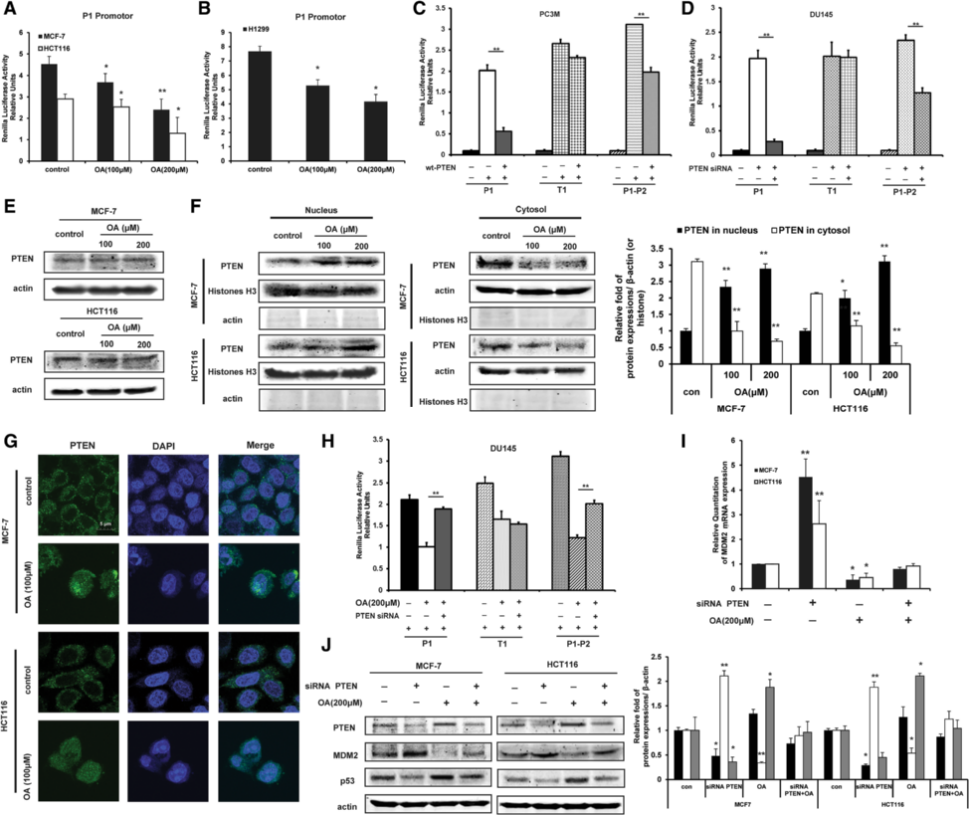
Oroxylin A inhibits the transcription of MDM2 via PTEN. **(A)** MCF-7 and HCT116 cells were transfected with an MDM2 promoter luciferase reporter plasmid (pGL3Basic-Mdm-P1-luc) and then treated with oroxylin A for 48 h. Luciferase activity was normalized to Renilla activity and expressed as luciferase/Renilla relative units. **(B)** H1299 cells were transfected with an MDM2 promoter luciferase reporter plasmid (pGL3Basic-Mdm-P1-luc) and then treated with oroxylin A for 48 h. Luciferase activity was measured. **(C)** Wt-PTEN plasmids were respectively co-transfected with MDM2 promoter luciferase reporter plasmids (pGL3Basic-Mdm-P1-luc, pGL3Basic-Mdm-T1-luc, or pGL3Basic-Mdm-P1-P2-luc) into PC3M cells. Luciferase activity was measured. **(D)** PTEN siRNA were respectively co-transfected with MDM2 promoter luciferase reporter plasmids (pGL3Basic-Mdm-P1-luc, pGL3Basic-Mdm-T1-luc, or pGL3Basic-Mdm-P1-P2-luc) into DU145 cells. Luciferase activity was measured. **(E)** Cells were treated with oroxylin A for 48 h. Western blot assays were performed for PTEN. **(F)** Nucleus and cytosolic fractions were isolated after treatment and subjected to Western blot analysis for PTEN. **(G)** Immunofluorescence experiment performed in MCF-7 and HCT116 cells upon oroxylin A treatment using antibodies specific to PTEN and DAPI. **(H)** PTEN siRNA were respectively co-transfected with MDM2 promoter luciferase reporter plasmids (pGL3Basic-Mdm-P1-luc, pGL3Basic-Mdm-T1-luc, or pGL3Basic-Mdm-P1-P2-luc) into DU145 cells. Cells were then treated with 200 μΜ oroxylin A for 48 h. Luciferase activity was measured. **(I)** MCF-7 and HCT116 cells were transfected with siRNA targeting PTEN or with a non-targeting control siRNA, then incubated with 200 μM oroxylin A for 48 h. The mRNA expression of MDM2 was detected by Quantitative RT-PCR. **(J)** Cells were transfected with siRNA targeting PTEN or with a non-targeting control siRNA and incubated with 200 μM oroxylin A for 48 h. Western blot assays were performed for p53, MDM2, and PTEN. All the Western Blot bands were quantified. Bars, SD; *p < 0.05 or **p < 0.01 versus non-treated control.

Our previous results suggested that oroxylin A could inhibit p53 degradation by downregulating MDM2 expression instead of influencing p53 mRNA level, which was mediated by SIRT3. The result that oroxylin A increased p53 levels suggested that oroxylin A must act through negative feedback on MDM2 transcription through the P2 promoter in wt-p53 cancer cells. However, the expression of SIRT3 still influenced the level of MDM2 in p53-null cells (Figure 4B). This inferred the possibility that oroxylin A may function via a p53-independent mechanism to regulate MDM2 transcription. For this purpose, a genomic DNA fragment containing the P1 promoter regions of the MDM2 gene only was ligated to a luciferase reporter gene. A luciferase assay showed that oroxylin A inhibited the transcription of MDM2 in wt-p53 MCF-7 and HCT116 cells (Figure 5A) as well as in p53-null H1299 cells (Figure 5B).

PTEN modulates MDM2 transcription by negatively regulating its P1 promoter [20]. Therefore, we tried to verify the direct modulation of PTEN on the MDM2 transcription by co-transfecting wt-PTEN plasmids with pGL3Basic-Mdm-P1-P2-luc, pGL3Basic-Mdm-P1-luc, or pGL3 Basic-Mdm-T1-luc (see the plasmid constructs in Additional file 1: Figure S2) in PTEN-null cell lines PC3M cells. As a result, wt-PTEN was co-transfected with the P1 promoter or P1-P2 promoter showed a significant increase in MDM2 transcriptional activity. Instead, wt-PTEN co-transfected with the T1 promoter had little effect (Figure 5C). Moreover, the deletion of PTEN decreased in the MDM2 transcriptional activity of wt-PTEN DU145 cells transfected with P1 promoter (Figure 5D).

Then, we investigated the effects of oroxylin A on PTEN. As shown in Figure 5E, oroxylin A had no significant effect on the expression of PTEN. However, we found that the protein level of PTEN in the cytosol decreased and instead increased in the nucleus (Figure 5F). And oroxylin A promoted the translocation of PTEN from the cytosol to the nucleus (Figure 5G). In further studies, deletion of PTEN reversed the oroxylin A-downregulated transcriptional activity of MDM2 in DU145 cells transfected with P1 promoter or P1-P2 promoter, instead having no effects in DU145 cells transfected with T1 promoter (Figure 5H). Moreover, siRNA-mediated silencing of PTEN reversed the oroxylin A-induced decrease of the mRNA and protein levels of MDM2 (Figure 5I, J). These results suggested that oroxylin A downregulated the mRNA expression of MDM2 via promoting PTEN-mediated negative transcription.

### SIRT3-mediated deacetylation increased PTEN lipid phosphatase activity, which was responsible for oroxylin A-induced negative regulation of MDM2 transcription

**Figure 6:**
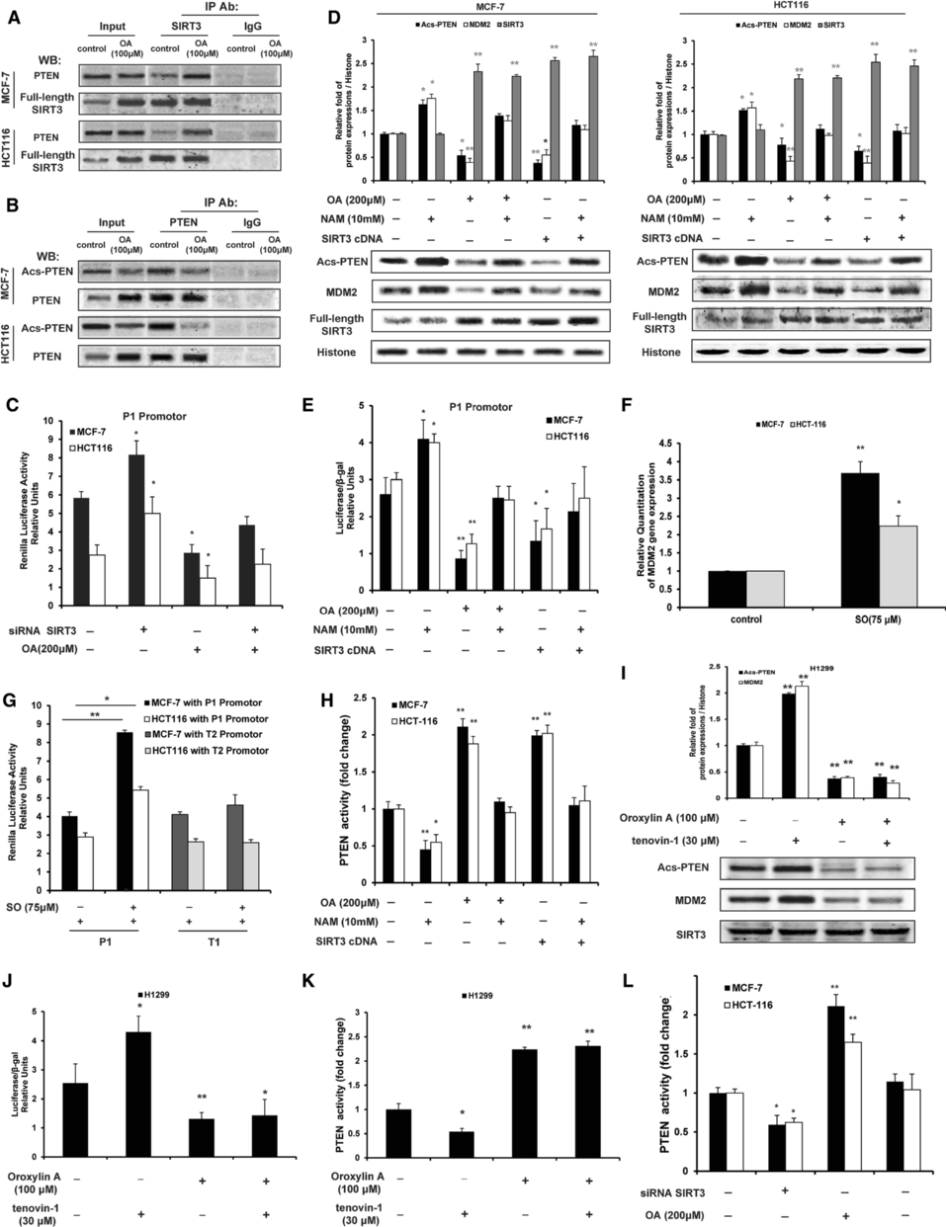
Oroxylin A inhibited transcription of MDM2 by promoting SIRT3-regulated lipid phosphatase activity of PTEN. **(A)** Cells were treated with oroxylin A (OA) for 48 h. Nuclei were isolated and PTEN was immunoprecipitated using anti-FL SIRT3 antibody. Western blot assays were performed for PTEN and FL SIRT3. **(B)** Nuclei were isolated and acetylated PTEN was immunoprecipitated using anti-PTEN antibody. Western blot assays were performed for acetylated-lysine and PTEN. **(C)** Cells were co-transfected with MDM2 promoter luciferase reporter plasmid (pGL3Basic-Mdm-P1-luc) and siRNA targeting SIRT3, then incubated with OA for 48 h. Luciferase activity was measured. **(D, E)** Cells were transfected with SIRT3 cDNA or treated with OA firstly. Then both were treated with NAM for 48 h. **(D)** Nuclei were isolated and Western blot assays were performed for MDM2, acetylated PTEN, and FL SIRT3. **(E)** Before treatments, MDM2 promoter luciferase reporter plasmid (pGL3Basic-Mdm-P1-luc) was co-transfected into cells. Luciferase activity was measured. **(F)** Cells were treated with sodium orthovanadate (SO) for 48 h. The mRNA expression of MDM2 was detected. **(G)** Cells were transfected with MDM2 promoter luciferase reporter plasmids (pGL3Basic-Mdm-P1-luc or pGL3Basic-Mdm-T1-luc), and then treated with SO for 48 h. Luciferase activity was measured. **(H)** Cells were treated as that in (D). Lipid phosphatase activity of PTEN was assayed. **(I, J, K)** H1299 cells were treated with OA in/without the presence of tenovin-1for 48 h. **(I)** Nuclei were isolated and Western blot assays were performed for MDM2 and acetylated PTEN. **(J)** Before treatment, cells were transfected with MDM2 promoter luciferase reporter plasmid (pGL3Basic-Mdm-P1-luc). Luciferase activity was measured. **(K)** Lipid phosphatase activity of PTEN was assayed. **(L)** Cells were transfected with siRNA targeting SIRT3 and incubated with OA for 48 h. Lipid phosphatase activity of PTEN was assayed. All the Western blot bands were quantified. Bars, SD; *p < 0.05 or **p < 0.01 versus non-treated control.

Human SIRT3 is expressed as a full-length 44-kDa protein and cleaved via the mitochondrial matrix processing peptidase (MPP) to a short 28-kDa protein, which is important for SIRT3 enzymatic activity [21]. However, the existence of a small pool of active nuclear SIRT3 has been proposed. This pool, consisting of the long form of SIRT3, has been suggested to have histone deacetylase activity [13]. Since the regulation of MDM2 levels by oroxylin A was mediated by SIRT3 and regulated by PTEN, we examined a potential link between PTEN and SIRT3. For this purpose, we assessed the binding between SIRT3 and PTEN by immunoprecipitation of nuclear proteins in MCF-7 and HCT116 cells. The results showed that full-length SIRT3 could bind with PTEN in the nucleus, and oroxylin A promoted this binding (Figure 6A). Moreover, oroxylin A decreased the acetylation of PTEN in MCF-7 and HCT116 cells (Figure 6B).

PTEN activity is regulated by acetylation, and the SIRT1 deacetylase is mainly responsible for PTEN deacetylation [22]. We then examined whether the negative transcriptional regulation of MDM2 effect via PTEN induced by oroxylin A was involved in the deacetylase activity of SIRT3. As shown in Figure 6C, siRNA-mediated silencing of SIRT3 promoted the transcription of the MDM2 gene and reversed the oroxylin A-induced suppression of MDM2 transcription. Moreover, overexpression of SIRT3 showed the same effects as oroxylin A, decreasing the acetylation of PTEN and the level of MDM2 as well as inhibiting the transcription of the MDM2 gene, whereas nicotinamide (NAM, SIRT inhibitor) reversed these effects (Figure 6D, E).

It has been reported that the transcriptional activity of PTEN is associated with its lipid phosphatase activity [20]. To investigate whether the transcription of MDM2 was related to PTEN lipid phosphatase activity, the lipid phosphatase inhibitor, sodium orthovanadate (SO), was used as a positive control [23]. As shown in Figure 6F, the gene expression of MDM2 was increased by SO in MCF-7 and HCT116 cells. As well, MCF-7 and HCT116 cells transfected with P1 promoter had increased transcription of MDM2 by SO (Figure 6G). Acetylation played a potential role in regulating PTEN function [22], which was mainly achieved lipid phosphatase activity. The lipid phosphatase activity of PTEN was increased by oroxylin A or overexpression of SIRT3, but decreased by NAM (Figure 6H).

The above results demonstrated that the SIRT3-mediated deacetylation of PTEN increased MDM2 transcription and PTEN lipid phosphatase activity. However, MNAM was the inhibitor of SIRT3 as well as SIRT1. Therefore, to make sure that the effects of oroxylin A can really be attributed to SIRT3-mediated, and not SIRT1-mediated deacetylation of PTEN, we used tenovin-1 for further investigation. Tenovin-1 was a small-molecule p53 activator, which inhibited SIRT1 and SIRT2 at low levels, as well as SIRT3 at higher levels [24]. Therefore, to avoiding the influence of p53, we used p53-null H1299 cells for investigation. As shown in Figure 6I–K, tenovin-1 inhibited deacetylation of PTEN, decreased the lipid phosphatase activity of PTEN, and suppressed the transcription of MDM2. When H1299 cells were treated with both tenovin-1 and oroxylin A, tenovin-1 had little influence on the effects of oroxylin A. Moreover, the increased lipid phosphatase activity by oroxylin A was reversed by the deletion of SIRT3 (Figure 6L). These results suggested that SIRT3, instead of SIRT1, played a critical role in the deacetylation of PTEN induced by oroxylin A, resulting in the promotion of PTEN lipid phosphatase activity, and the decreased transcription of MDM2.

### The deacetylation of PTEN mediated by oroxylin A played important roles in the regulation of glycolysis in cancer cells

**Figure 7:**
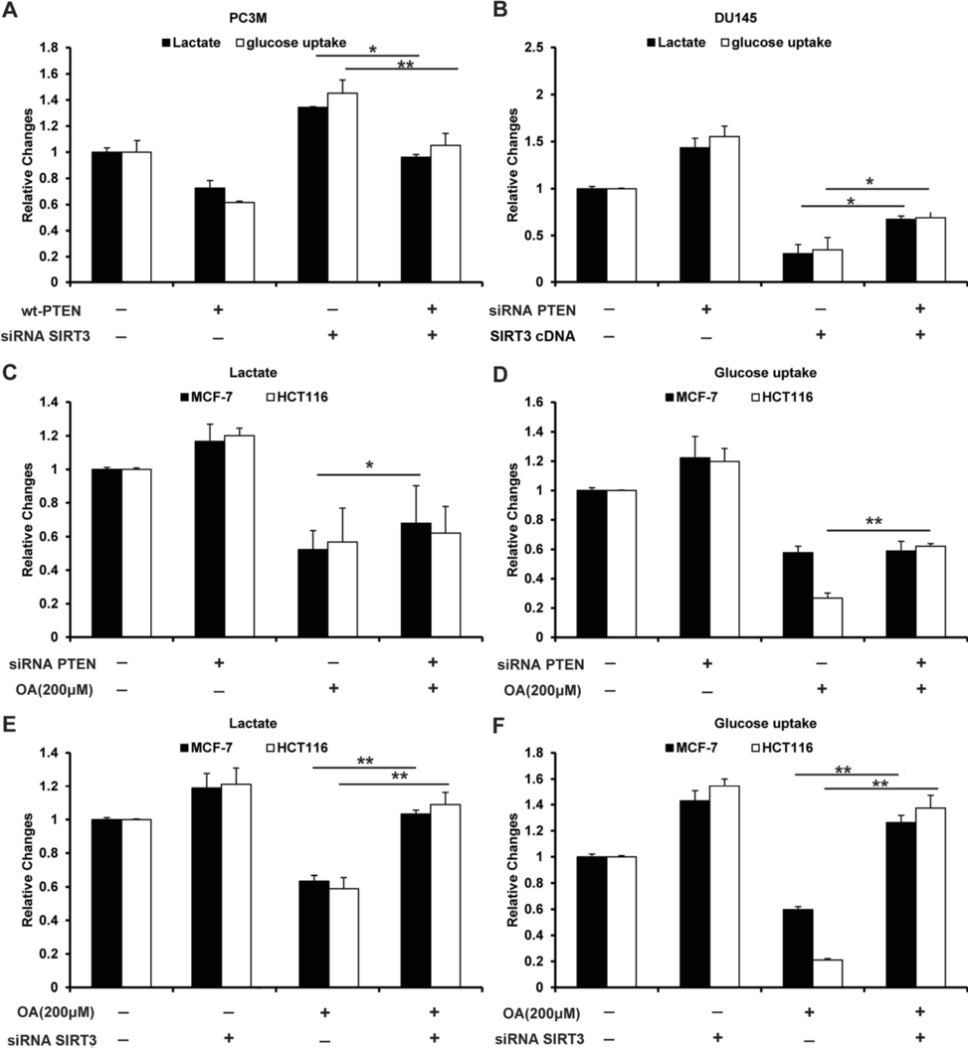
Oroxylin A-regulated glycolysis involved in the deacetylation of PTEN. **(A)** PC3M cells were co-transfected with wt-PTEN plasmids and siRNA SIRT3. Glucose uptake and lactate production were detected. **(B)** DU145 cells were co-transfected with PTEN siRNA and SIRT3 cDNA. Glucose uptake and lactate production were detected. **(C, D)** MCF-7 and HCT116 cells were transfected with siRNA targeting PTEN or with a non-targeting control siRNA, then incubated with 200 μM oroxylin A for 48 h. Lactate production **(E)** and glucose uptake **(F)** were detected. **(E, F)** MCF-7 and HCT116 cells were transfected with siRNA SIRT3, and then treated with 200 μM oroxylin A for 48 h, Lactate production **(E)** and glucose uptake **(F)** were detected. Bars, SD; *p < 0.05 or **p < 0.01.

In our studies, we had found that oroxylin A opposed glycolysis via p53 and stabilized p53, which was resulted from the PTEN-regulated negative transcription of MDM2. Moreover, the deacetylation of PTEN increased the transcriptional activity of PTEN. Certainly, PTEN affects glycolysis [25], but the role of acetylation is not well defined. In our further studies, by overexpressing wt-PTEN in PTEN-null cell lines PC3M cells or knocking out PTEN in wt-PTEN DU145 cells, we found the expression of PTEN inhibited glycolysis, and the deletion of deacetylase SIRT3 promoted glycolysis (Figure 7A, B). Although the deletion of PTEN in MCF-7 and HCT116 cells reversed the inhibition of glycolysis by oroxylin A (Figure 7C, D), the inhibition of deacetylated PTEN by siRNA SIRT3 had more strong influence than the suppression of PTEN expression on the oroxylin A-regulated glycolysis (Figure 7E, F). These results showed that the deacetylation of PTEN mediated by oroxylin A played important roles in glycolysis.

### Oroxylin A inhibited the growth of nude mice xenograft tumor-inoculated MCF-7 and HCT116 cells *in vivo* by downregulating MDM2 level and p53-regulated glycolytic proteins

**Figure 8:**
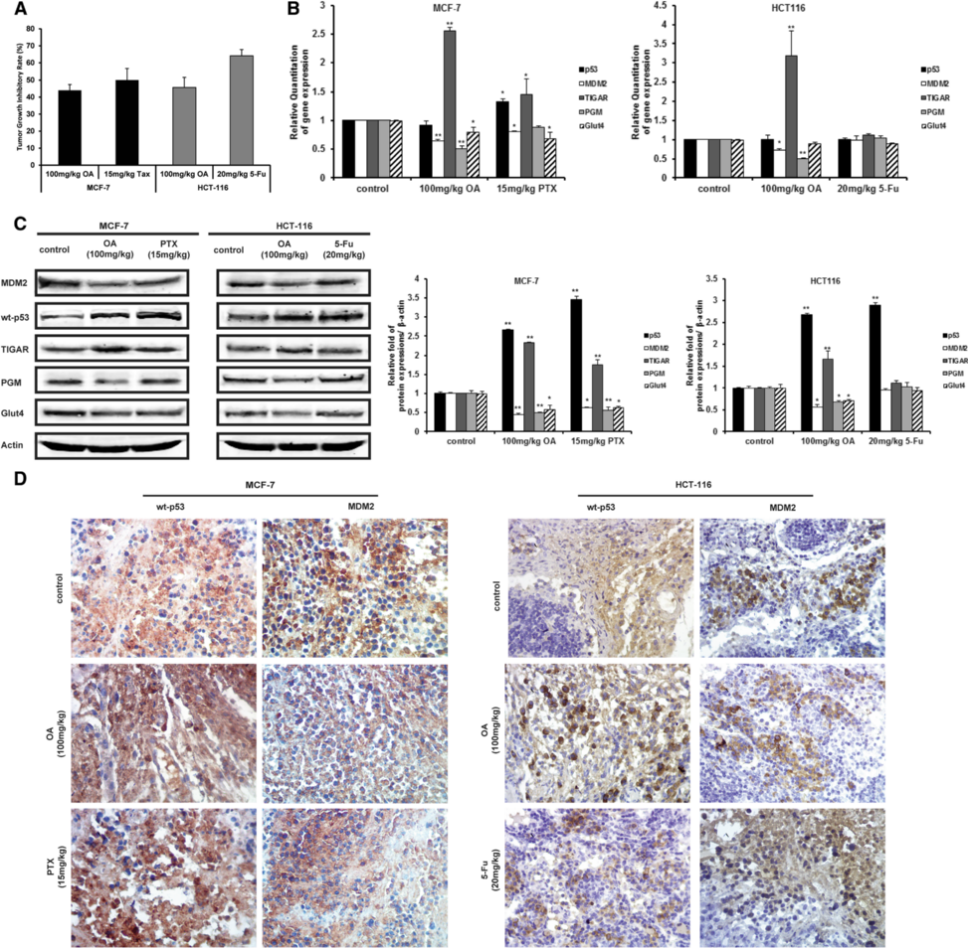
Oroxylin A inhibited the growth-transplanted human tumor. Nude mice inoculated with MCF-7 cells were treated with saline control, oroxylin A (100 mg/kg), and PTX (15 mg/kg). Nude mice inoculated with HCT116 cells were treated with saline control, oroxylin A (100 mg/kg), and 5-FU (20 mg/kg). **(A)** The tumor inhibitory rates were calculated. **(B)** Quantitative RT-PCR on RNA isolated from xenograft tumors. **(C)** The tumor tissue protein expressions of xenograft tumors were assayed by immunoblotting. **(D)** Proteins expression in breast tumor tissue were assessed by immunohistochemistry. All the Western blot bands were quantified. Bars, SD; *p < 0.05 or **p < 0.01 versus non-treated control.

We performed xenograft experiment with HCT-116 or MCF-7 cells. As shown in Figure 8A, the inhibitory effect of 100 mg/kg oroxylin A on tumor growth of HCT-116 or MCF-7 cells were 45.65% and 43.95%, respectively. The inhibitory effect of oroxylin A on HCT116 cells was a little weaker than 5 Fu (20 mg/kg, the inhibitory rate was 64.14%) and was as strong as that of paclitaxel (PTX) (15 mg/kg, the inhibitory rate was 50.05%) on MCF-7 cells. The tissue extracted from the tumor samples of nude mice were used for gene and protein expression assay. As shown in Figure 8B–D, oroxylin A increased p53 protein expression in MCF-7 and HCT116 cells and had little effects on its gene expression, while both the protein level and gene level of MDM2 were decreased by oroxylin A. Moreover, the protein and gene level of p53-targeting glycolytic enzyme were changed accordingly, PGM and GLUT4 were decreased, and TIGAR was increased (Figure 8B, C).

These data suggested that the inhibition of oroxylin A on xenograft tumors of HCT-116 or MCF-7 cells were aroused by the suppression of p53-mediated glycolysis in some degree.

## Discussion

**Figure 9:**
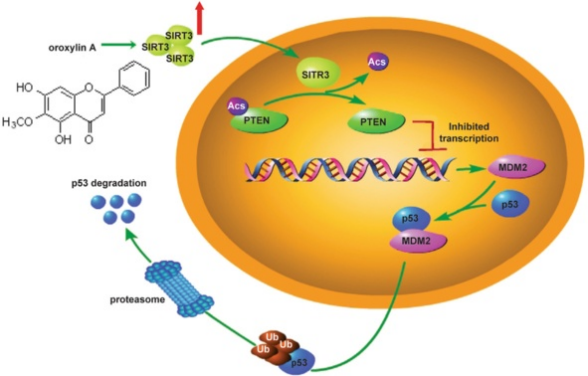
Schematic diagram describing the effect of oroxylin A on the inhibition of p53 degradation through the suppression of PTEN-regulated MDM2 expression. Oroxylin A enhanced cellular SIRT3 level, causing the deacetylation of PTEN and promoting its lipid phosphatase activity. The activated PTEN suppressed the transcription of MDM2, which was responsible for the degradation of p53. As a result, oroxylin A inhibited p53 degradation and the p53-related glycolytic pathway.

As a hallmark of cancer, the Warburg effect, which consists of the activation of aerobic glycolysis, provides pathologists and clinicians clues to diagnose cancer and helps to explain how cancerous processes prepare substrates to support rapid cell growth. p53, which is considered a critical ‘node’ of the cellular circuitry, plays important roles in the metabolic shift of cancer cells by influencing several aspects of metabolism through different mechanisms. In general, p53 suppresses aerobic glycolysis and promotes mitochondrial respiration through the transcriptional regulation of target genes, providing a mechanism for blocking tumorigenesis [4,26]. Here, we investigated the mechanisms underlying the effect of oroxylin A on the regulation of p53 and p53-related glycolytic pathways. We found that the oroxylin A inhibited the MDM2-mediated p53 degradation and glycolysis in wt-p53 cancer cells. And oroxylin A had a stronger inhibitory effect on glycolysis in wt-p53 cancer cells than in mut-p53 cancer cells (Additional file 2: Figure S1A to Additional file 2: Figure S1D). Furthermore, oroxylin A repressed the PTEN-mediated transcription of MDM2 by promoting its SIRT3-mediated deacetylation (Figure 9).

The p53 pathway is frequently disrupted in tumor cells. Therefore, recovering the function of wild-type p53 and its targets in tumor cells is a significant therapeutic objective. A small-molecule compound, RITA (p53 activator III), was reported to inhibit glycolytic enzymes and induce robust apoptosis in cancer cells [27]. In addition to the pharmacological activation of wild-type p53, such as the effect of RITA, increasing the stability of the p53 protein is another strategy for restoring wild-type p53 activity in cancer cells. The protein level of wild-type p53 is regulated by the HDM2 ubiquitin ligase, which targets p53 for degradation by catalyzing its ubiquitination. HDM2 inhibitors such as Nutlin 3A can stabilize p53 and rescue its tumor suppressor function in cancer cells [28]. However, the antitumor efficacy of agents that promote a functional p53 is often accompanied by adverse effects [29,30]. Nutlin 3A carries the risk of enhancing the prosurvival adaptation functions of p53 in some tumors, promoting the p53-dependent upregulation of Notch1 and triggering a negative feedback anti-apoptotic mechanism [31]. In the present study, oroxylin A upregulated p53 protein level by inhibiting the MDM2-mediated degradation (Figure 3). Notably, oroxylin A showed the potential to overcome the drug resistance caused by the p53-dependent upregulation of factors that promote the growth of cancer cells. Oroxylin A affects different cellular pathways and functions as an anticancer drug via multiple effects, including the induction of apoptosis and cell cycle arrest, the inhibition of angiogenesis, the suppression of invasion and metastasis, and the reversal of multidrug-resistance [32-36]. Therefore, despite the activation of p53-dependent cancer-promoting factors, oroxylin A acts by counteracting cancer-promoting effects through the activation of different pathways and it modulates p53 levels to promote its anticarcinogenic effects.

Our results showed that oroxylin A inhibited the MDM2-dependent degradation of wt-p53. The stabilization and transcriptional activation of wt-p53 in response to various stresses is crucial for cellular homeostasis. Oroxylin A inhibited glycolysis by regulating the transcription of the p53 target genes TIGAR, PGM, and GLUT4, but did not influence the transcriptional level of p53 (Figure 2C). In mut-p53 cells, oroxylin A did not influence mut-p53 level and showed different effects on the level of TIGAR, PGM, and GLUT4 with those in wt-p53 cells (Additional file 2: Figure S1E and Additional file 2: Figure S1F). Our results indicated that oroxylin A affected the post-transcriptional regulation of p53. Ubiquitination, phosphorylation, and acetylation are post-translational modifications that affect the levels and activity of p53. Recent findings suggested that these modifications had a profound effect on p53 stability and function [37]. Our previous studies suggested that SIRT3-mediated deacetylation played an important role in oroxylin A-induced suppression of glycolysis. The inhibition of cellular deacetylases leads to a longer half-life for endogenous p53 [38]. SIRT1 is the homologous protein of SIRT3 in the SIRT family, and negatively regulates the tumor suppressor p53 [39]. Therefore, we considered the possibility that SIRT3 may play a similar role as SIRT1. Our results showed that wt-p53 as well as mut-p53 could not bind with SIRT3, and oroxylin A had no effect on their interaction (Figure 3F and Additional file 2: Figure S1I). However, oroxylin A increased SIRT3 protein level in both wt-p53 cancer cells and mut-p53 cancer cells (Figure 4A and Additional file 2: Figure S1G). We found that oroxylin A inhibited p53 degradation via a different mechanism related with SIRT3 instead of influencing the direct action between p53 and SIRT3.

In addition to the modification of p53, MDM2 can be modified post-transcriptionally to disrupt the p53-MDM2 interaction. Similar to p53, the activity of MDM2 can be regulated by acetylation [40]. Oroxylin A could not inhibit the binding of p53 with MDM2 (Figure 3D). However, the transcription of MDM2 was significantly suppressed by oroxylin A (Figures 2C and 3B). The human MDM2, HDM2, is controlled by two different promoters [41,42]. Transcription from the first promoter, P1, is independent of p53, whereas transcription from the second promoter, P2, is p53-dependent. As shown in Figure 4B, oroxylin A decreased the level of MDM2 in null-p53 cancer cells, suggesting that it modulates MDM2 levels in a p53-independent manner. Therefore, we constructed a luciferase reporter gene consisting of the P1 promoter DNA fragment of the MDM2 gene only and showed that oroxylin A could inhibit the transcriptional activity of MDM2 through a p53-independent mechanism (Figure 5A, B). *In vivo*, we showed that oroxylin A inhibited the tumor growth of wt-p53 MCF-7 and HCT116 cells (Figure 8). And the inhibitory rate of 100 mg/kg oroxylin A for mut-p53 MDA-MB-231 cells was 56.78% (data not shown) and 36.16% for HT29 cells [16]. These results suggested that oroxylin A had the presence of a p53-independent mechanism as well.

The PTEN tumor suppressor gene is a major tumor suppressor that physically interacts with p53 and prevents its degradation by dissociating p53 from the p53-MDM2 complex [43,44]. The overexpression of wt-PTEN in the PTEN-null cell lines PC3M cells, which were co-transfected with pGL3Basic-Mdm-P1-luc, promoted the transfection of MDM2, instead in the PTEN-null cell lines PC3M cells pGL3 Basic-Mdm-T1-luc not (Figure 5C). Oroxylin A could promote the translocation of PTEN from the cytosol to the nucleus (Figure 5G). Professor Wu and his group reported a novel mechanism by which PTEN modulates MDM2 expression independent of p53 [20]. Our results showed that the deletion of PTEN reversed the oroxylin A-induced suppression of MDM2 transcription (Figure 5H, I). PTEN was shown to control MDM2 P1 promoter activity through its lipid phosphatase activity.

The lipid phosphatase activity of PTEN is critical for its tumor suppressor function [45]. In addition to phosphorylation and membrane association, which regulate PTEN activity, acetylation is an important mechanism of regulation of PTEN function and it involves the activity of SIRT1 [22]. The deacetylation of the PTEN, as well as its expression, both influenced glycolysis in cancer cells (Figure 7). Moreover, we found that SIRT3 induced the deacetylation of PTEN similar to SIRT1 (Figure 6A, B). The dependence of p53 level and MDM2 transcription on SIRT3 highlights the significance of SIRT3 in the anticancer effects of oroxylin A (Figures 4C and 6C). To make sure whether the effects of oroxylin A were attributed to SIRT3-mediated, and not SIRT1-mediated deacetylation of PTEN, p53-null H1299 cells were treated with both tenovin-1 and oroxylin A, and found that tenovin-1 had little influence on the effects of oroxylin A (Figure 6I–K). Therefore, SIRT3 played a critical role in the deacetylation of PTEN induced by oroxylin A, but not SIRT1. Oroxylin A upregulated the lipid phosphatase activity of PTEN via SIRT3-mediated deacetylation. Interestingly, our results show that the SIRT3-mediated deacetylation of PTEN occurs in the nucleus rather than in the mitochondria. Determining the subcellular localization of SIRT3 is crucial for identifying its targets and substrates, and elucidating its cellular functions is crucial as well for the identification of its associated signaling pathways [21]. Although most studies support a mitochondrial localization and deacetylase activity for SIRT3 [46,47], others report that both forms of SIRT3 are enzymatically active [13]. Prof. Reinberg Iwahara et al. report that SIRT3 is capable of histone deacetylase (HDAC) activity and that the full-length (FL) SIRT3 is associated with transcriptional repression dependent on its HDAC activity [48]. Our findings that FL SIRT3 in the nucleus induced the deacetylation of PTEN suggest that oroxylin A regulates MDM2 transcription by promoting the *deacetylation* of PTEN.

## Conclusions

Previous studies have shown that the flavonoid oroxylin A increases p53 levels and inhibits p53-mediated glycolysis [17,49]. In the present study, we showed that oroxylin A inhibited glycolysis in wt-p53 cancer cells through the suppression of p53 degradation. PTEN-mediated suppression of MDM2 transcription is responsible for the increased p53 level. And the lipid phosphatase activity of PTEN was regulated by the FL SIRT3-mediated deacetylation, playing the key roles in the effects of oroxylin A on p53. Further understanding of the effects of oroxylin A on key glycolytic regulatory factors may help reveal critical mechanisms for the design of treatments targeting cancer metabolism.

## Materials and methods

### Reagents

Oroxylin A (C_16_H_12_O_5_, purity 99.76%, the synthetic route, structure assay and purity assay; see Additional file 3: Figure S3 and Additional file 4: Table S1) was isolated from the root of *Scutellaria baicalensis* Georgi, according to previously reported protocols [50], dissolved in DMSO as a stock solution at −20°C, and diluted with a medium before each experiment. The final DMSO concentration did not exceed 0.1% throughout the study. SRT 1720 hydrochloride (SRT1720) was purchased from Santa Cruz Biotechnology (Santa Cruz, CA), dissolved in DMSO, and prepared to 10^−1^ M stock solutions. CHX, MG132 (proteasome inhibitor), and NAM were purchased from Beyotime (Beyotime Institute of BioTechnology, Haimen, China). Tenovin-1 was obtained from Cayman Chemical Co. (Ann Arbor, MI) and diluted to a 10^−1^ M concentration in DMSO.

### Cell Culture

The human breast cancer cell lines MDA-MB-231 and MCF-7 and the human colon cancer cell lines HCT-116 and HT-29 were purchased from Cell Bank of Shanghai Institute of Biochemistry and Cell Biology, Chinese Academy of Sciences (Shanghai, China). MCF-7 cells were cultured in and Dulbecco’s MEM (DMEM, Invitrogen Corporation, Carlsbad, CA); HCT-116 was cultured in McCoy’s 5A Medium (GIBCO, Invitrogen Corporation, Carlsbad, CA), both supplemented with 10% heat-inactivated fetal bovine serum (Sijiqing, Hangzhou, China), 100 U/ml penicillin G, and 100 μg/ml streptomycin at 37°C, 95% relative humidity, and 5% CO_2_ with 21% oxygen conditions.

### Lactic acid production

To measure lactic acid production, cells were treated with oroxylin A for 48 h, and media were collected and assayed following the manufacturer’s instructions of the Lactic Acid production Detection kit (KeyGen, Nanjing, China). The assay results were detected with a spectrophotometer (Thermo, Waltham, MA) at 570 nm.

### Glucose uptake assay

After treatment, media were collected and diluted 1:4000 in water. The amount of glucose in the media was then detected using the Amplex Red Glucose Assay Kit (Invitrogen, Eugene, OR) according to the manufacturer’s instructions. Glucose uptake was determined by subtracting the amount of glucose in each sample from the total amount of glucose in the media (without cells). The detection was performed by spectrophotometer (Thermo, Waltham, MA) at Ex/Em = 530/590 nm.

### Western blot analysis

Protein samples were isolated with lysis buffer, eluted with SDS buffer, separated on SDS-polyacrylamide gels, and electroblotted onto PVDF membranes [51]. Immunoreactive protein bands were detected using an Odyssey Scanning System (LI-COR Inc., Superior St., Lincoln, NE). The following antibodies were used for Western blotting: PGM, MDM2, β-actin (Santa Cruz Biotechnology, CA) at 1:400 dilution; SIRT3, hexokinase II, hydroxy-HIF-1α, PTEN, GLUT4 (Cell signaling Technology, Inc., MA) at 1:800 dilution; SIRT3 of nuclear protein (Abcam Ltd, HK, China) at 1:1000 dilution; TIGAR (Anspec, Inc., San Jose, CA); p53 (Ab-6) (EMD Chemicals, Gibbstown, NJ).

### Real-time PCR analysis

Total RNA was extracted using the TriPure Isolation Reagent (Roche Diagnostics, Mannheim, Germany) and then amplified by polymerase chain reaction (PCR). An aliquot of 1 μg of total RNA was used to transcribe the first-strand cDNA with SuperScript II reverse transcriptase (Invitrogen, Eugene, OR). Real-time PCR was completed on an ABI PRISM Sequence Detector 7500 (PerkinElmer, Branchburg, NJ) using Sequence Detector version 1.7 software (Applied Biosystems, Foster City, CA). SYBR Green PCR Master Mix was purchased from Applied Biosystems. The primer sets used in the PCR amplification were listed in Table 1. The relative gene expressions were analyzed using quantitative RT-PCR with β-actin as an internal control.

**Table 1:** **The primer sequence used in the PCR amplification**

**Gene**	**Sense**	**Antisense**
β-actin	5′-CTGTCCCTGTATGCCTCTG-3′	5′-ATGTCACGCACGAT-TTCC-3′
p53	5′-CTCCTCAGCATCTTATCCG-3′	5′-AGCCTGGGCATCCTTG-3′
MDM2	5′-CTTGATGCTGGTGTAAGT-3′	5′-GTTGATGGCTGAGAATAG-3′
TIGAR	5′-CAGTGATCTCATGAGGACAAAGCA-3′	5′-CCATGGCCCTCAGCTCACTTA-3′
PGM	5′-TTGAATACA GCGACCCAGTGGA-3′	5′-CTATCGATGTACAGCCGAATGGTG-3′
GLUT4	5′-CTTCATCATTGGCATGGGTTT-3′	5′-AGGACCGCAAATAGAAGGAAGA-3′

### Immunoprecipitation

SIRT3 was immunocaptured using antibodies against SIRT3 cross-linked to protein G-agarose beads (Santa Cruz Biotechnology, CA). The immunocomplexes were analyzed by Western blotting and probed with antibodies against p53 (Ab-6) and MDM2.

Wt-p53 was immunocaptured using p53 (Ab-6) cross-linked to protein G-agarose beads (Santa Cruz Biotechnology, CA). The immunocomplexes were analyzed by Western blotting and probed with antibody against MDM2.

Full-length SIRT3 was immunocaptured from nuclear extracts using antibodies against full-length SIRT3 (Abcam Ltd., HK, China) cross-linked to protein G-agarose beads. The PTEN protein was analyzed by Western blotting and probed with anti-PTEN antibody.

PTEN was immunocaptured from nuclear extracts using antibodies against PTEN cross-linked to protein G-agarose beads. The acetylated PTEN was analyzed by Western blotting and probed with acetylated-lysine antibody.

### Cell transfection and luciferase reporter assay

The MDM2 luciferase reporter gene plasmid pGL3Basic-Mdm-P1-P2-luc, pGL3Basic-Mdm-P1-luc, and pGL3Basic-Mdm-T1-luc were designed according to the studies of Prof. Hong Wu (Dept. of Molecular and Medical Pharmacology, Howard Hughes Medical Institute, Los Angeles, CA) [20] and synthesized by Beyotime Institute of BioTechnology (Hangzhou, China) (Additional file 1: Figure S2). Cells (5 × 10^5^ cells/well) were plated in 6-well plates and transfected transiently with the pGL3Basic-Mdm-P1-luc containing the P1 promoter of MDM2 only using Lipofectamine 2000TM reagent (Invitrogen, CA). The plasmid GL3Basic-Mdm-P1-luc was added to adjust the total amount of DNA (4 μg/well in a 6-well plate) and the Renilla luciferase reporter at 0.4 μg/well in a 6-well plate served as normalization control. Cells were treated with oroxylin A for 48 h and luciferase assays were performed with the Luciferase Reporter Gene Assay kit (Promega, Madison, WI) and detected using Luminoskan ascent (Thermo, Waltham, MA).

### Plasmid and siRNA transient transfection

The pCMV-Neo-Bam p53 plasmid containing the complete sequence of human wt-p53 and the pCMV-Neo-Bam p53 R249S plasmid containing the sequence of human mut-p53 were a gift from Prof. Moshe Oren (The Weizmann Institute of Science, Rehovot, Israel) and obtained from Addgene. The siRNAs targeting sirtuin-3 or sirtuin-3 cDNA were purchased from OriGene (OriGene Technologies, Inc., MD, USA). The siRNAs targeting PTEN were purchased from Santa Cruz Biotechnology (Santa Cruz, CA).

For siRNA transfection, cells were seeded in 6-well plates. Either p53 siRNA duplexes (30 pmol/l) or PTEN siRNA was introduced into the cells using siPORT NeoFX Transfection Agent (Ambion Inc., Austin, TX) according to the manufacturer’s recommendations. Then, the cells were exposed to RPMI 1640 medium with or without oroxylin A and harvested for further experiments.

For plasmid transfection, plasmid DNA (1 μg) was introduced using PolyJet In Vitro DNA Transfection Reagent (SignaGen Laboratories, Rockville, MD) according to the manufacturer’s recommendations. Cells were then exposed to oroxylin A or the vehicle and harvested for further experiments.

### Preparation of nuclear- and cytosol-enriched extracts

After cells were incubated with oroxylin A for 48 h, cell nuclear and cytoplasmic fractions were prepared using a nuclear/cytosol fractionation kit of Biovision Inc. (Mountain View, CA, USA) according to the manufacture’s direction.

### Immunofluorescence and confocal fluorescence microscopy

Cells were fixed with 4% paraformaldehyde in PBS at 1-h intervals, permeabilized with 0.5% Triton X-100, and blocked with 3% BSA for 30 min. Incubation with primary antibodies against PTEN (Bioworld Technology, Inc, MN, USA) was done overnight at 4°C. Then, the nuclei were stained with 4′,6-diamidino-2-phenylindole (DAPI, Sigma-Aldrich) 20 min before imaging. A laser scanning confocal microscope FV10-ASW (Ver 2.1) (Olympus Corp, MPE FV1000, Tokyo, Japan) was used for colocalization analysis.

### PTEN lipid phosphatase activity

For the measurement of *in vitro* PTEN lipid phosphatase activity, the malachite green phosphatase assay kit (Echelon Biosciences, Inc., Salt Lake City, UT) was used according to the manufacturer’s instructions (see the detailed process in Additional file 5).

### *In vivo* tumor growth assay

This experiment was conducted in accordance with the guidelines issued by the State Food and Drug Administration (SFDA of China).

Twenty nude mice were inoculated subcutaneously with 1 × 10^7^ HCT-116 into the right axilla. After 12 days of growth, tumor sizes were determined using micrometer calipers. Mice-inoculated HCT-116 cells with similar tumor volumes were randomly divided into the following three groups (six mice/group): saline control, oroxylin A (100 mg/kg, i.v., every 2 days), and 5-Fluorouracil (5-Fu, 20 mg/kg, i.v., every 2 days).

To facilitate estrogen-dependent xenograft establishment, each mouse received 17-estradiol (20 mg/kg; Sigma) intraperitoneally once a week. One week after treatment, equivalent amounts of MCF-7 cells were injected subcutaneously (10^7^ cells/tumor) into the left axilla of nude mice. After 12 days of growth, tumor sizes were determined using micrometer calipers. Mice-inoculated MCF-7 cells with similar tumor volumes were randomly divided into the following three groups (six mice/group): saline control, oroxylin A (100 mg/kg, i.v., every 2 days), and PTX (15 mg/kg, i.v., twice a week).

Tumor sizes were measured every 3 days using micrometer calipers, and tumor volume was calculated using the following formula: TV (mm3) = d^2^ × D/2, where d and D were the shortest and the longest diameters, respectively. Mice were sacrificed on day 21, and tumor tissues were used for Western blotting, real-time PCR, and Immunohistochemistry Assay (see the detailed process in Additional file 5).

### Statistical evaluation

Data are presented as mean ± SD from triplicate parallel experiments unless otherwise indicated. Statistical analyses were performed using one-way ANOVA.

## Additional files

Additional file 1: Figure S2. Schematic illustration of MDM2 promoter and reporter constructs. P1, promoter 1, P2, promoter 2; P1-P2, MDM2 full-length promoter region including both promoters; T1, a serial 5′ truncated Mdm2 promoter construct.

Additional file 2: Figure S1. The effect of oroxylin A on SIRT3, MDM2, mut-p53, and p53-related glycolytic pathway in mut-p53 cancer cells. (A, B) wt-p53 cancer cells (MCF-7 and HCT116) and mut-p53 (MDA-MB-231 and HT-29 cells) were treated with oroxylin A (100 and 200 μΜ) for 48 h. (A) Glucose uptake was measured using the Amplex Red assay. (B) Production of lactic acid was assayed by Lactic Acid production Detection kit. (C, D) MDA-MB-231 and HT-29 were transfected with siRNA targeting wt-p53 or with a non-targeting control siRNA, then incubated with 200 μM oroxylin A for 48 h. Glucose uptake (C) and lactate production (D) were detected. (E) MDA-MB-231 and HT-29 cells were treated with oroxylin A (100 and 200 μΜ) for 48 h. Western blot assays were performed for the p53-targeted gene products p53, TIGAR, PGM, and GLUT4. (F) H1299 cells were transfected with a cDNA clone targeting mut-p53 (R248W) or with a non-targeting vector, and then incubated with 200 μM oroxylin A for 48 h. Western blot assays were performed for the p53-targeted gene products TIGAR, PGM, and GLUT4. (G) Western blot assays were performed for the MDM2 and SIRT3. (H) MDM2 was immunoprecipitated using p53 (Ab3) antibodies. Western blot assays were performed for MDM2, mut-p53. (I) p53 (Ab3) was immunoprecipitated using anti-SIRT3 antibody. Western blot assays were performed for mut-p53 and SIRT3. All the Western Blot bands were quantified.

Additional file 3: Figure S3. The detailed information of oroxylin A. (A) The structure and molecular weight of oroxylin A. (B) The synthetic route of oroxylin A. In the synthesis, baicalein is used as the starting material, and participated in benzyl reaction to compound (2), which is methylated to produce compounds (3). Then compound (3) was participated in palladium hydrogen/carbon reduction reaction to get the target product oroxylin A. (C) NMR assay for the structure of isolated sample. 1H-NMR spectra were determined on a Varian Gemini-300 NMR instrument. (D) MS assay for the structure of isolated sample. Mass spectra were recorded on a Finnigan MAT TSQ-46 or Finnigan MAT TSQ-700 mass spectrometer. The data was listed as below: ^1^H-NMR (DMSO-d_6_, 300Hz): δ3.85 (3H, s, OMe), 6.63 (1H, s, 3H), 6.95 (1H, S, 8H), 7.56-7.59 (3H, m, ArH), 8.05-8.07 (2H, d, ArH), 10.78 (1H, s, 7-OH), 12.92 (1H, s, 5-OH). MS (EI, m/z): 284 (MH1). IR (KBr,υ) cm^-1^:1653, 3455. (E) IR assay for the structure of isolated sample. IR spectra were recorded on a Perkin-Elmer FT-IR 1600 series FT-IR spectrophotometer. (F) The sample was analyzed by HPLC.

Additional file 4: Table S1. The HPLC analysis for purity of oroxylin A.

Additional file 5: **Supplementary materials and methods.** The methods for animal model, PTEN lipid phosphatase activity, and immunohistochemistry were listed.

## Abbreviations

SIRT3: Sirtuin-3
PTEN: Phosphatase and tensin homolog
MDM2: Mouse double minute 2
SCO2: Cytochrome c oxidase 2
TIGAR: TP53-induced glycolysis and apoptosis regulator
PGM: Phosphoglycerate mutase
GLUT: Glucose transporter
OA: Oroxylin A
SRT1720: SRT 1720 hydrochloride
CHX: Cycloheximide
NAM: Nicotinamide

## References

[1] Vousden KH, Prives C (2009). Blinded by the light: the growing complexity of p53. Cell.

[2] Vogelstein B, Lane D, Levine AJ (2000). Surfing the p53 network. Nature.

[3] Levine AJ (1997). p53, the cellular gatekeeper for growth and division. Cell.

[4] Puzio-Kuter AM (2011). The role of p53 in metabolic regulation. Genes Cancer.

[5] Schwartzenberg-Bar-Yoseph F, Armoni M, Karnieli E (2004). The tumor suppressor p53 down-regulates glucose transporters GLUT1 and GLUT4 gene expression. Cancer Res.

[6] Bensaad K, Tsuruta A, Selak MA, Vidal MN, Nakano K, Bartrons R (2006). TIGAR, a p53-inducible regulator of glycolysis and apoptosis. Cell.

[7] Matoba S, Kang JG, Patino WD, Wragg A, Boehm M, Gavrilova O (2006). p53 regulates mitochondrial respiration. Science.

[8] Kondoh H, Lleonart ME, Gil J, Wang J, Degan P, Peters G (2005). Glycolytic enzymes can modulate cellular life span. Cancer Res.

[9] Itahana K, Mao H, Jin A, Itahana Y, Clegg HV, Lindstrom MS (2007). Targeted inactivation of Mdm2 RING finger E3 ubiquitin ligase activity in the mouse reveals mechanistic insights into p53 regulation. Cancer Cell.

[10] Ryan KM, Phillips AC, Vousden KH (2001). Regulation and function of the p53 tumor suppressor protein. Curr Opin Cell Biol.

[11] Saunders LR, Verdin E (2007). Sirtuins: critical regulators at the crossroads between cancer and aging. Oncogene.

[12] Kim HS, Patel K, Muldoon-Jacobs K, Bisht KS, Aykin-Burns N, Pennington JD (2010). SIRT3 is a mitochondria-localized tumor suppressor required for maintenance of mitochondrial integrity and metabolism during stress. Cancer Cell.

[13] Scher MB, Vaquero A, Reinberg D (2007). SirT3 is a nuclear NAD + -dependent histone deacetylase that translocates to the mitochondria upon cellular stress. Genes Dev.

[14] Wei L, Zhou Y, Dai Q, Qiao C, Zhao L, Hui H (2013). Oroxylin A induces dissociation of hexokinase II from the mitochondria and inhibits glycolysis by SIRT3-mediated deacetylation of cyclophilin D in breast carcinoma. Cell Death Dis.

[15] Qiao C, Wei L, Dai Q, Zhou Y, Yin Q, Li Z (2015). UCP2-related mitochondrial pathway participates in oroxylin A-induced apoptosis in human colon cancer cells. J Cell Physiol.

[16] Ha J, Zhao L, Zhao Q, Yao J, Zhu BB, Lu N (2012). Oroxylin A improves the sensitivity of HT-29 human colon cancer cells to 5-FU through modulation of the COX-2 signaling pathway. Biochem Cell Biol.

[17] Mu R, Qi Q, Gu H, Wang J, Yang Y, Rong J (2009). Involvement of p53 in oroxylin A-induced apoptosis in cancer cells. Mol Carcinog.

[18] Mayo LD, Donner DB (2001). A phosphatidylinositol 3-kinase/Akt pathway promotes translocation of Mdm2 from the cytoplasm to the nucleus. Proc Natl Acad Sci U S A.

[19] Zhang YY, Zhou LM (2012). Sirt3 inhibits hepatocellular carcinoma cell growth through reducing Mdm2-mediated p53 degradation. Biochem Biophys Res Commun.

[20] Chang CJ, Freeman DJ, Wu H (2004). PTEN regulates Mdm2 expression through the P1 promoter. J Biol Chem.

[21] Alhazzazi TY, Kamarajan P, Verdin E, Kapila YL (2011). SIRT3 and cancer: tumor promoter or suppressor?. Biochim Biophys Acta.

[22] Ikenoue T, Inoki K, Zhao B, Guan KL (2008). PTEN acetylation modulates its interaction with PDZ domain. Cancer Res.

[23] Aaltonen N, Lehtonen M, Varonen K, Goterris GA, Laitinen JT (2012). Lipid phosphate phosphatase inhibitors locally amplify lysophosphatidic acid LPA1 receptor signalling in rat brain cryosections without affecting global LPA degradation. BMC Pharmacol.

[24] Lain S, Hollick JJ, Campbell J, Staples OD, Higgins M, Aoubala M (2008). Discovery, in vivo activity, and mechanism of action of a small-molecule p53 activator. Cancer Cell.

[25] Ortega-Molina A, Serrano M (2013). PTEN in cancer, metabolism, and aging. Trends Endocrinol Metab.

[26] Shen L, Sun X, Fu Z, Yang G, Li J, Yao L (2012). The fundamental role of the p53 pathway in tumor metabolism and its implication in tumor therapy. Clin Cancer Res.

[27] Zawacka-Pankau J, Grinkevich VV, Hunten S, Nikulenkov F, Gluch A, Li H (2011). Inhibition of glycolytic enzymes mediated by pharmacologically activated p53: targeting Warburg effect to fight cancer. J Biol Chem.

[28] Elison JR, Cobrinik D, Claros N, Abramson DH, Lee TC (2006). Small molecule inhibition of HDM2 leads to p53-mediated cell death in retinoblastoma cells. Arch Ophthalmol.

[29] Lu C, El-Deiry WS (2009). Targeting p53 for enhanced radio- and chemo-sensitivity. Apoptosis.

[30] Yang ZX, Wang D, Wang G, Zhang QH, Liu JM, Peng P (2010). Clinical study of recombinant adenovirus-p53 combined with fractionated stereotactic radiotherapy for hepatocellular carcinoma. J Cancer Res Clin Oncol.

[31] Bertheau P, Espie M, Turpin E, Lehmann J, Plassa LF, Varna M (2008). TP53 status and response to chemotherapy in breast cancer. Pathobiology.

[32] Li HN, Nie FF, Liu W, Dai QS, Lu N, Qi Q (2009). Apoptosis induction of oroxylin A in human cervical cancer HeLa cell line in vitro and in vivo. Toxicology.

[33] Yang Y, Hu Y, Gu HY, Lu N, Liu W, Qi Q (2008). Oroxylin A induces G2/M phase cell-cycle arrest via inhibiting Cdk7-mediated expression of Cdc2/p34 in human gastric carcinoma BGC-823 cells. J Pharm Pharmacol.

[34] Gao Y, Lu N, Ling Y, Chen Y, Wang L, Zhao Q (2010). Oroxylin A inhibits angiogenesis through blocking vascular endothelial growth factor-induced KDR/Flk-1 phosphorylation. J Cancer Res Clin Oncol.

[35] Sun Y, Lu N, Ling Y, Gao Y, Chen Y, Wang L (2009). Oroxylin A suppresses invasion through down-regulating the expression of matrix metalloproteinase-2/9 in MDA-MB-435 human breast cancer cells. Eur J Pharmacol.

[36] Yang HY, Zhao L, Yang Z, Zhao Q, Qiang L, Ha J (2012). Oroxylin A reverses multi-drug resistance of human hepatoma BEL7402/5-FU cells via downregulation of P-glycoprotein expression by inhibiting NF-kappaB signaling pathway. Mol Carcinog.

[37] Brooks CL, Gu W (2003). Ubiquitination, phosphorylation and acetylation: the molecular basis for p53 regulation. Curr Opin Cell Biol.

[38] Ito A, Lai CH, Zhao X, Saito S, Hamilton MH, Appella E (2001). p300/CBP-mediated p53 acetylation is commonly induced by p53-activating agents and inhibited by MDM2. EMBO J.

[39] Yi J, Luo J (2010). SIRT1 and p53, effect on cancer, senescence and beyond. Biochim Biophys Acta.

[40] Wang X, Taplick J, Geva N, Oren M (2004). Inhibition of p53 degradation by Mdm2 acetylation. FEBS Lett.

[41] Juven T, Barak Y, Zauberman A, George DL, Oren M (1993). Wild type p53 can mediate sequence-specific transactivation of an internal promoter within the mdm2 gene. Oncogene.

[42] Zauberman A, Flusberg D, Haupt Y, Barak Y, Oren M (1995). A functional p53-responsive intronic promoter is contained within the human mdm2 gene. Nucleic Acids Res.

[43] Freeman DJ, Li AG, Wei G, Li HH, Kertesz N, Lesche R (2003). PTEN tumor suppressor regulates p53 protein levels and activity through phosphatase-dependent and -independent mechanisms. Cancer Cell.

[44] Zhou M, Gu L, Findley HW, Jiang R, Woods WG (2003). PTEN reverses MDM2-mediated chemotherapy resistance by interacting with p53 in acute lymphoblastic leukemia cells. Cancer Res.

[45] Myers MP, Pass I, Batty IH, Van der Kaay J, Stolarov JP, Hemmings BA (1998). The lipid phosphatase activity of PTEN is critical for its tumor supressor function. Proc Natl Acad Sci U S A.

[46] Cooper HM, Spelbrink JN (2008). The human SIRT3 protein deacetylase is exclusively mitochondrial. Biochem J.

[47] Bao J, Lu Z, Joseph JJ, Carabenciov D, Dimond CC, Pang L (2010). Characterization of the murine SIRT3 mitochondrial localization sequence and comparison of mitochondrial enrichment and deacetylase activity of long and short SIRT3 isoforms. J Cell Biochem.

[48] Iwahara T, Bonasio R, Narendra V, Reinberg D (2012). SIRT3 functions in the nucleus in the control of stress-related gene expression. Mol Cell Biol.

[49] Dai Q, Yin Y, Liu W, Wei L, Zhou Y, Li Z (2013). Two p53-related metabolic regulators, TIGAR and SCO2, contribute to oroxylin A-mediated glucose metabolism in human hepatoma HepG2 cells. Int J Biochem Cell Biol.

[50] Li HB, Chen F (2005). Isolation and purification of baicalein, wogonin and oroxylin A from the medicinal plant Scutellaria baicalensis by high-speed counter-current chromatography. J Chromatogr A.

[51] Zhao L, Guo QL, You QD, Wu ZQ, Gu HY (2004). Gambogic acid induces apoptosis and regulates expressions of Bax and Bcl-2 protein in human gastric carcinoma MGC-803 cells. Biol Pharm Bull.

