# Correction to: Oroxylin A promotes PTEN-mediated negative regulation of MDM2 transcription via SIRT3-mediated deacetylation to stabilize p53 and inhibit glycolysis in wt-p53 cancer cells

**DOI:** 10.1186/s13045-019-0792-8

**Published:** 2019-12-30

**Authors:** Kai Zhao, Yuxin Zhou, Chen Qiao, Ting Ni, Zhiyu Li, Xiaotang Wang, Qinglong Guo, Na Lu, Libin Wei

**Affiliations:** 1 State Key Laboratory of Natural Medicines, Jiangsu Key Laboratory of Carcinogenesis and Intervention, China Pharmaceutical University, 24 Tongjiaxiang, Nanjing, 210009 People’s Republic of China; 2 Department of Medicinal Chemistry, China Pharmaceutical University, 24 Tongjiaxiang, Nanjing, 210009 People’s Republic of China; 3 Department of Chemistry and Biochemistry, Florida International University, Miami, FL 33199 USA

**Correction to: J Hematol Oncol**

**https://doi.org/10.1186/s13045-015-0137-1**

The original article [1] contains several errors:

1. During WB experiment, authors conducted multiple batches of samples together, and thus confused the actin for different batches of samples. When organising the results, authors misused the incorrect WB band for actin of MCF7 cells in Fig. 2a as that of HCT116 cells in Fig. 2d. Authors would like to correct the actin protein band in Fig. 2d and correct version is shown ahead.
2. During WB experiment, authors conducted the samples for HCT116 cells in Fig. 3a and the samples for HCT116 cells in Fig. 3b together in the same gel. When organising the results, authors made a mistake with the actin protein band and erroneously used the actin protein band repetitively. The actin for Fig. 3b is correct. Authors would like to correct the actin protein band in Fig. 3a, and correct version is shown ahead.
3. The resolution and quality for some WB bands in Fig. 3d and e are low, resulting in potential misunderstanding. Authors would like to replace the low quality WB bands with a higher quality version shown ahead.
4. To avoid confusion, authors would like to clarify that actin bands shown in Figs. 3b and 4a are from the same gel in a one experiment. Thus, the actin protein bands for them are same.

**Fig. 2:**
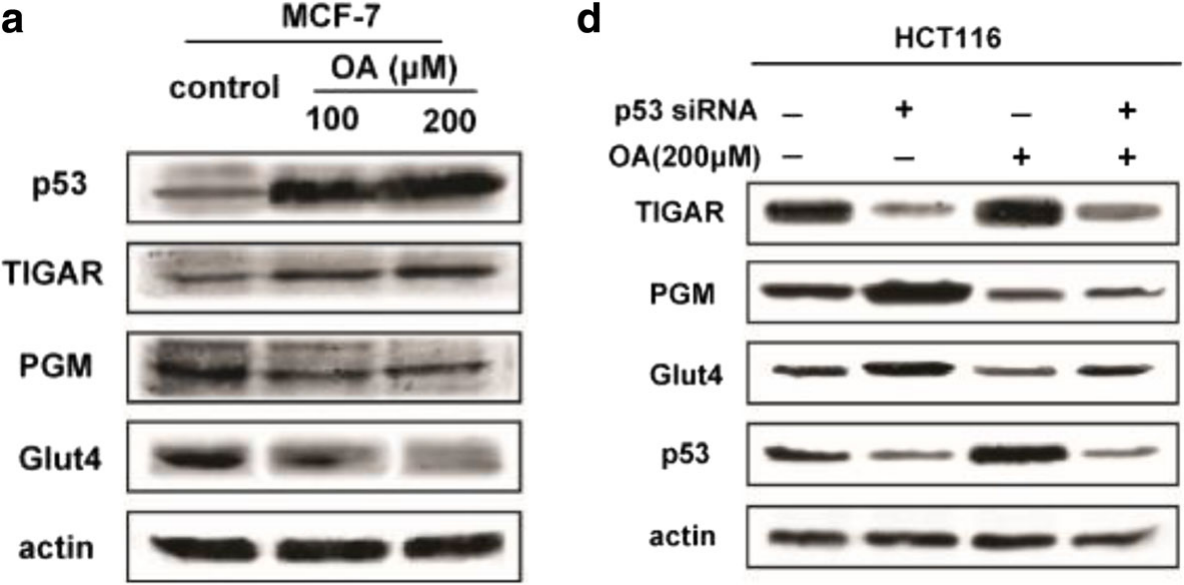
Oroxylin A downregulates the protein and mRNA expression of p53-related glycolytic pathway components. (**a**) MCF-7 and HCT116 cells were treated with oroxylin A (100 and 200 μM) for 48 h. Western blot assays were performed for the p53-targeted gene products p53, TIGAR, PGM, and GLUT4. (**d**) MCF-7 and HCT116 were transfected with siRNA targeting wt-p53 or with a non-targeting control siRNA, then incubated with 200 μM oroxylin A for 48 h. Western blot assays were performed for the p53-targeted gene products TIGAR, PGM, and GLUT4. All the Western Blot bands were quantified. Bars, SD;*p<0.05 or **p<0.01 versus non-treated control

**Fig. 3:**
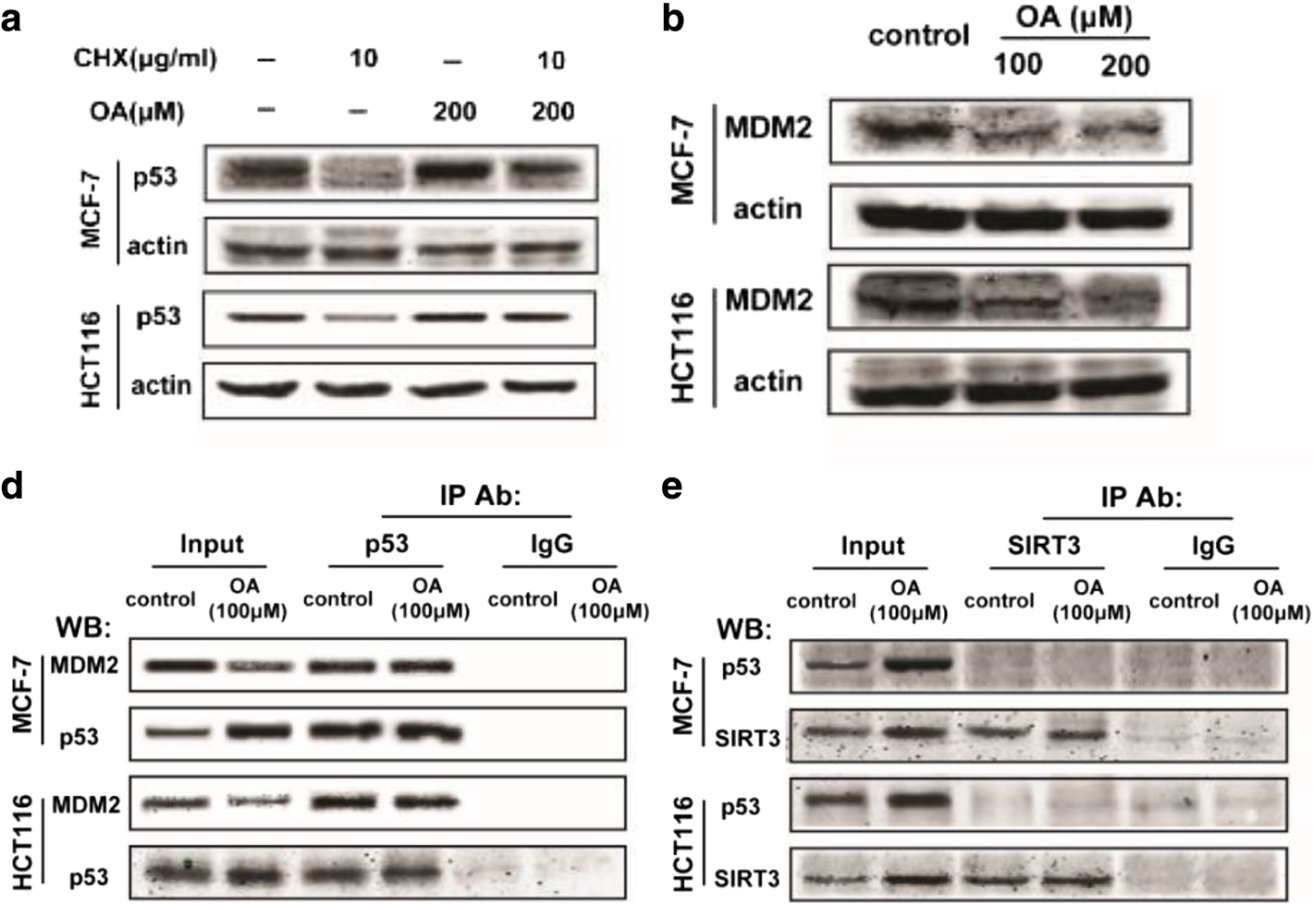
Oroxylin A enhances p53 expression through post-transcriptional regulation. (**a**) Cells were treated with oroxylin A (100 and 200 μM) for 48 h. Western blot assays were performed for MDM2. (**b**) Effect of oroxylin A on p53 expression after co-treatment with CHX. Cells were treated with vehicle or oroxylin A for 48 h, and 6 h before harvested, 10 μg/ml CHX was added to the medium. p53 protein expression was detected by Western blotting. (**d**) MDM2 was immunoprecipitated using p53 (Ab6) antibodies. Western blot assays were performed for MDM2, p53. (**e**) p53 (Ab6) was immunoprecipitated using anti-SIRT3 antibody. Western blot assays were performed for p53 and SIRT3. All the Western blot bands were quantified
