# Supplementary figures and images for: Oroxylin A promotes PTEN-mediated negative regulation of MDM2 transcription via SIRT3-mediated deacetylation to stabilize p53 and inhibit glycolysis in wt-p53 cancer cells
Source: J Hematol Oncol. 2015 Apr 23;8:41. doi: 10.1186/s13045-015-0137-1 (PMC4419472; doi:10.1186/s13045-015-0137-1)

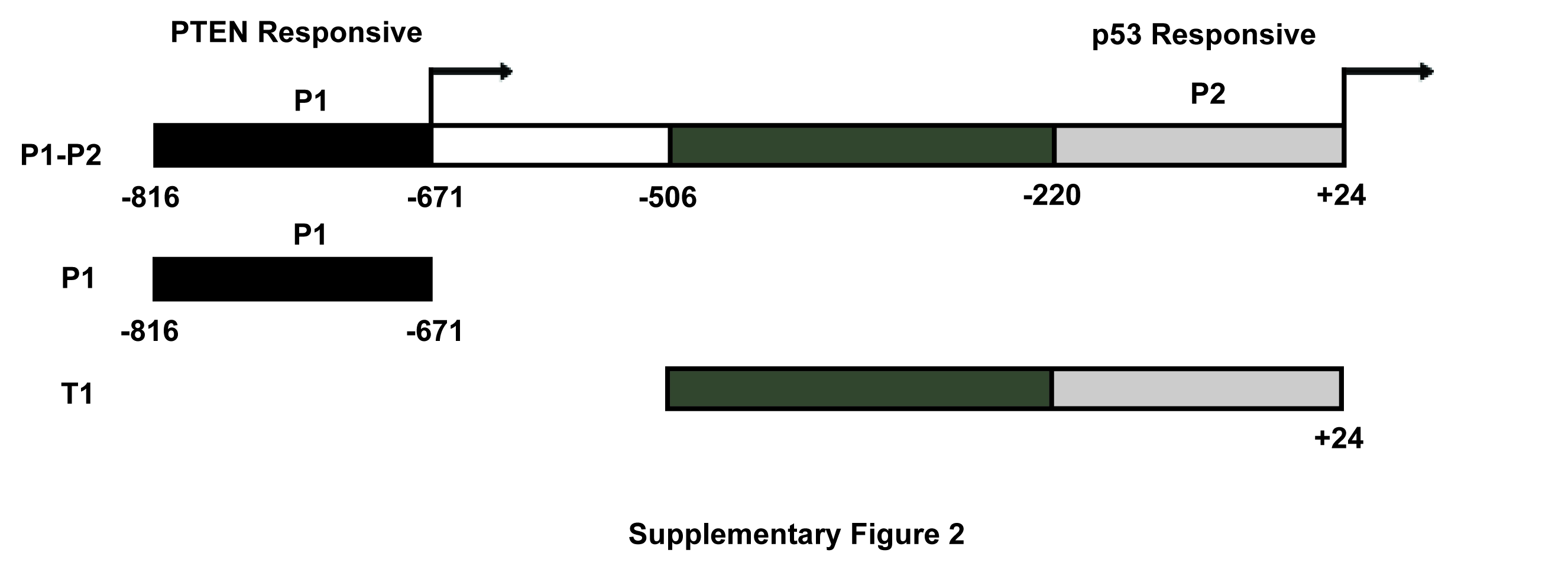

Supplement: Supplementary file 1 — Schematic illustration of MDM2 promoter and reporter constructs. P1, promoter 1, P2, promoter 2; P1-P2, MDM2 full-length promoter region including both promoters; T1, a serial 5′ truncated Mdm2 promoter construct. [file 13045_2015_137_MOESM1_ESM.tiff]

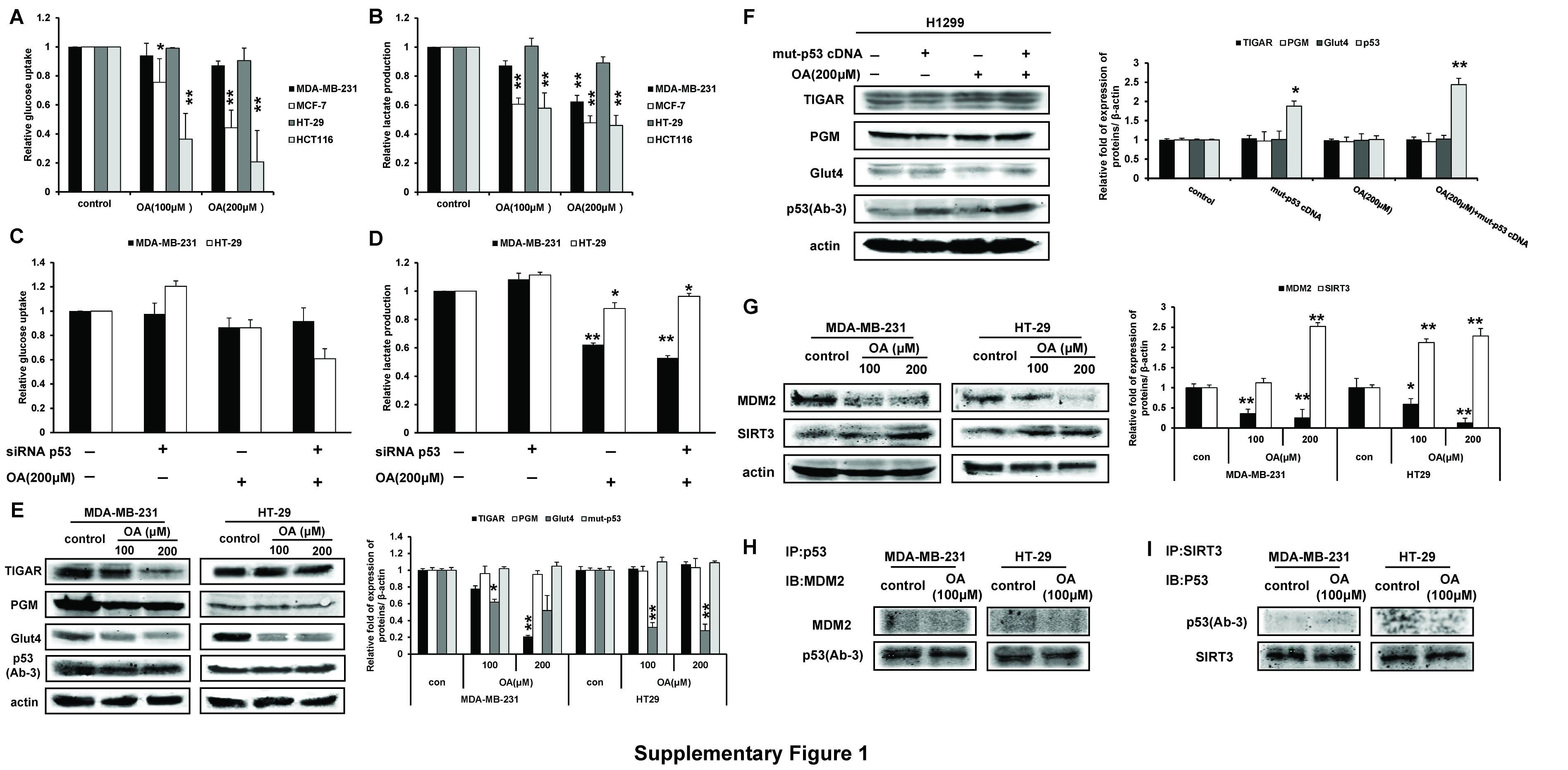

Supplement: Supplementary file 2 — The effect of oroxylin A on SIRT3, MDM2, mut-p53, and p53-related glycolytic pathway in mut-p53 cancer cells. (A, B) wt-p53 cancer cells (MCF-7 and HCT116) and mut-p53 (MDA-MB-231 and HT-29 cells) were treated with oroxylin A (100 and 200 μΜ) for 48 h. (A) Glucose uptake was measured using the Amplex Red assay. (B) Production of lactic acid was assayed by Lactic Acid production Detection kit. (C, D) MDA-MB-231 and HT-29 were transfected with siRNA targeting wt-p53 or with a non-targeting control siRNA, then incubated with 200 μM oroxylin A for 48 h. Glucose uptake (C) and lactate production (D) were detected. (E) MDA-MB-231 and HT-29 cells were treated with oroxylin A (100 and 200 μΜ) for 48 h. Western blot assays were performed for the p53-targeted gene products p53, TIGAR, PGM, and GLUT4. (F) H1299 cells were transfected with a cDNA clone targeting mut-p53 (R248W) or with a non-targeting vector, and then incubated with 200 μM oroxylin A for 48 h. Western blot assays were performed for the p53-targeted gene products TIGAR, PGM, and GLUT4. (G) Western blot assays were performed for the MDM2 and SIRT3. (H) MDM2 was immunoprecipitated using p53 (Ab3) antibodies. Western blot assays were performed for MDM2, mut-p53. (I) p53 (Ab3) was immunoprecipitated using anti-SIRT3 antibody. Western blot assays were performed for mut-p53 and SIRT3. All the Western Blot bands were quantified. [file 13045_2015_137_MOESM2_ESM.tiff]

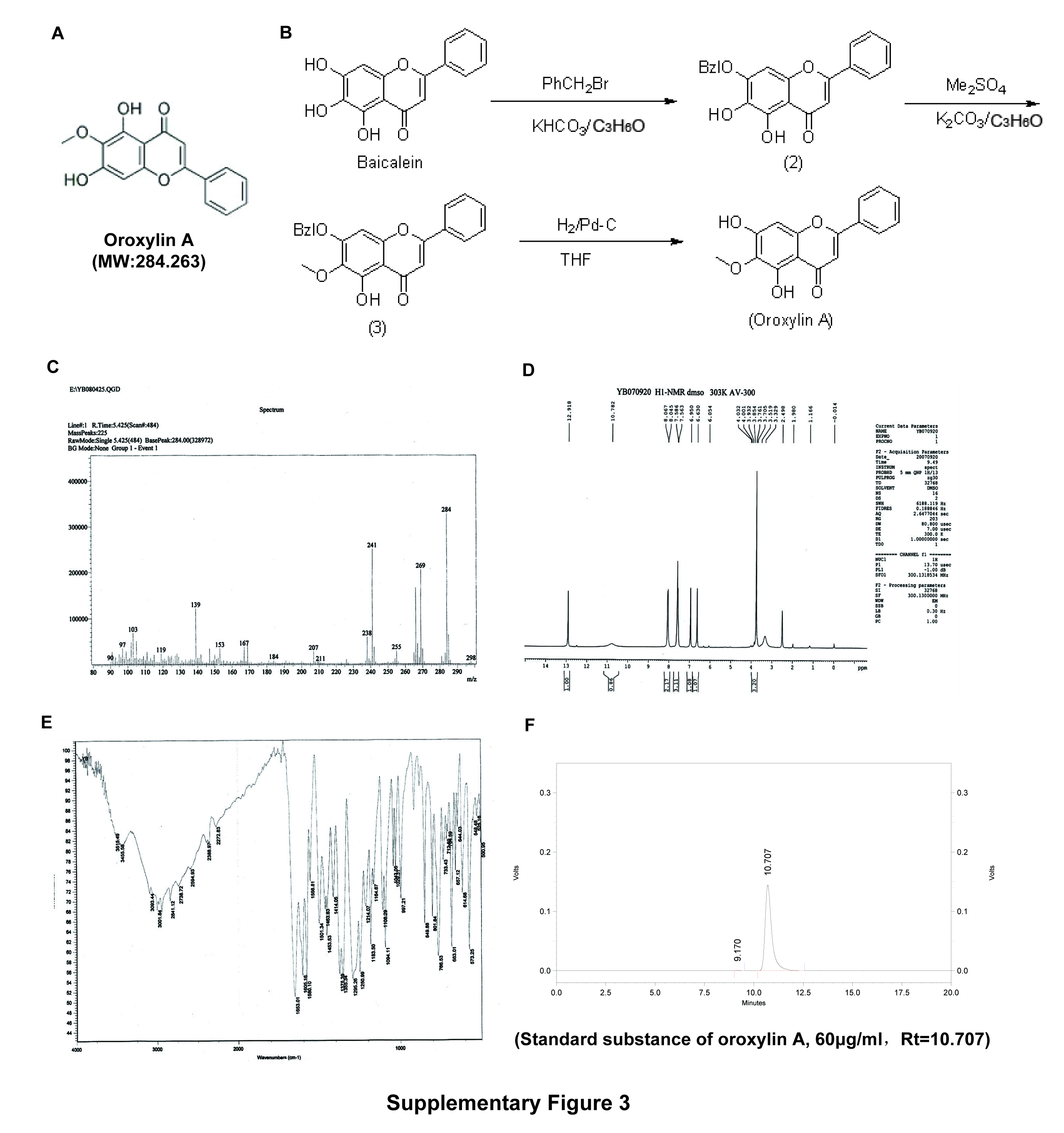

Supplement: Supplementary file 3 — The detailed information of oroxylin A. (A) The structure and molecular weight of oroxylin A. (B) The synthetic route of oroxylin A. In the synthesis, baicalein is used as the starting material, and participated in benzyl reaction to compound (2), which is methylated to produce compounds (3). Then compound (3) was participated in palladium hydrogen/carbon reduction reaction to get the target product oroxylin A. (C) NMR assay for the structure of isolated sample. 1H-NMR spectra were determined on a Varian Gemini-300 NMR instrument. (D) MS assay for the structure of isolated sample. Mass spectra were recorded on a Finnigan MAT TSQ-46 or Finnigan MAT TSQ-700 mass spectrometer. The data was listed as below: 1H-NMR (DMSO-d6, 300Hz): δ3.85 (3H, s, OMe), 6.63 (1H, s, 3H), 6.95 (1H, S, 8H), 7.56-7.59 (3H, m, ArH), 8.05-8.07 (2H, d, ArH), 10.78 (1H, s, 7-OH), 12.92 (1H, s, 5-OH). MS (EI, m/z): 284 (MH1). IR (KBr,υ) cm-1:1653, 3455. (E) IR assay for the structure of isolated sample. IR spectra were recorded on a Perkin-Elmer FT-IR 1600 series FT-IR spectrophotometer. (F) The sample was analyzed by HPLC. [file 13045_2015_137_MOESM3_ESM.tiff]
