# Supplementary material for: Oroxylin A promotes PTEN-mediated negative regulation of MDM2 transcription via SIRT3-mediated deacetylation to stabilize p53 and inhibit glycolysis in wt-p53 cancer cells
Source: J Hematol Oncol. 2015 Apr 23;8:41. doi: 10.1186/s13045-015-0137-1 (PMC4419472; doi:10.1186/s13045-015-0137-1)
Supplement: Supplementary file 4 — The HPLC analysis for purity of oroxylin A. [file 13045_2015_137_MOESM4_ESM.doc]

Supplemental Table 1. The HPLC analysis for purity of oroxylin A.

| Detector A (275nm) |  |  |  |  |  |
| --- | --- | --- | --- | --- | --- |
| **Pk #** | **Retention Time** | **Area** | **Area %** | **Height** | **Height Percent** |
| 1 | 9.170 | 5097 | 0.150 | 353 | 0.24 |
| 2 | 10.707 | 3385660 | 99.850 | 144528 | 99.76 |
| Totals |  | 3390757 | 100.000 | 144881 | 100.00 |
