# Supplementary material for: Oroxylin A promotes PTEN-mediated negative regulation of MDM2 transcription via SIRT3-mediated deacetylation to stabilize p53 and inhibit glycolysis in wt-p53 cancer cells
Source: J Hematol Oncol. 2015 Apr 23;8:41. doi: 10.1186/s13045-015-0137-1 (PMC4419472; doi:10.1186/s13045-015-0137-1)
Supplement: Supplementary file 5 — Supplementary materials and methods. The methods for animal model, PTEN lipid phosphatase activity, and immunohistochemistry were listed. [file 13045_2015_137_MOESM5_ESM.doc]

**Supplementary Materials and methods**

**Animal model.** Female athymic BALB/c nude mice (35–40 days old) with body weight ranging from 18 to 22 g were supplied by the Academy of Military Medical Sciences of the Chinese People’s Liberation Army (Certificate No. SCXK-(Army) 2007-004). The animals were kept at 22±2 °C and 55−65% humidity in stainless steel cages under controlled lights (12 h light/day) and were fed with standard laboratory food and water. Animal care was conducted in accordance with the recommendations of the Guide for the Care and Use of Laboratory Animals published by the National Institute of Health, USA.

**PTEN lipid phosphatase activity.** For the measurement of in vitro PTEN lipid phosphatase activity, the malachite green phosphatase assay kit (Echelon Biosciences, Inc., Salt Lake City, UT) was used according to the manufacturer's instructions. Briefly, 500 μg of cell lysate was subjected to PTEN immunoprecipitation by the addition of 8 μl anti-PTEN antibody (Santa Cruz Biotechnology, Santa Cruz, CA), and the immunocomplex formed was captured by incubation with 20 μl protein A/G beads for 3.5 h with gentle rotation at 4°C. The beads were then washed twice in lysis buffer and once in enzyme reaction buffer (ERB) (50 mM Tris-HCl, pH 8.0, 50 mM NaCl, 10 mM dithiothreitol, and 10 mM MgCl_2_) and resuspended in 80 μl prewarmed (37°C) ERB and distributed in triplicates of 20 μl in a 96-well flat-bottom plate (Echelon, CA). The reaction was initiated by adding 30 μl of ERB containing the substratedioctanoyl phosphatidylinositol 3,4,5-trisphosphate (PIP3-DiC8) (Echelon, CA) to 10 μM final concentration; it was left for 1 h at 37°C and stopped by 100 μl malachite green solution (Echelon, CA), and the absorbance was read at 620 nm after 15 min of incubation at room temperature. A PIP3-only blank was used in parallel to correct for potential nonspecific phosphate release. The remaining beads were used for SDS-PAGE, Western blotting, and densitometric quantification to confirm that equivalent amounts of PTEN were immunoprecipitated from all samples. A standard curve was made by using the phosphate solution provided with the kit. The PTEN activity was expressed as pmol phosphate released into the solution per hour per 20 μl of sample.

**Immunohistochemistry.** The expression of wt-p53 and MDM2 in the tissues of the control and treated groups was assessed by the SP immunohistochemical method using a rabbit-antihuman monoclonal antibody and an Ultra-Sensitive SP kit (kit 9710 MAIXIN, Maixin-Bio Co., Fujian, China). Tissue sections (4 mm thick) were placed onto treated slides (Vectabond, Vector Laboratories, Burlingame, CA, USA). Sections were heat fixed, deparaffinized and rehydrated through a graded alcohol series (100%, 95%, 85%, 75%) to distilled water. Tissue sections were boiled in citrate buffer at high temperature for antigen retrieval, and treated with 3% hydrogen peroxide to block endogenous peroxidase activity. The slides were incubated with a protein-blocking agent (kit 9710 MAIXIN, Maixin-Bio Co., Fuzhou, Fujian) prior to the application of the primary antibody, and then incubated with the primary antibody at 4°C overnight. The tissues were then incubated with the secondary biotinylated antispecies antibody and labeled using a modification of the avidin–biotin complex immunoperoxidase staining procedure according to the UltraSensitive SP kit manual. Counterstaining was done with Harris hematoxylin.
